# Intranasal delivery of engineered extracellular vesicles loaded with miR-206-3p antagomir ameliorates Alzheimer's disease phenotypes

**DOI:** 10.7150/thno.103596

**Published:** 2024-11-04

**Authors:** Dong Peng, Tingting Liu, Huahui Lu, Lei Zhang, Hongxia Chen, Yadong Huang, Bo Hu, Qihao Zhang

**Affiliations:** 1 State Key Laboratory of Bioactive Molecules and Druggability Assessment, Guangdong Basic Research Center of Excellence for Natural Bioactive Molecules and Discovery of Innovative Drugs, College of Life Science and Technology, Jinan University, Guangzhou 510632, China.; 2 Department of Cell Biology & Institute of Biomedicine, College of Life Science and Technology, Jinan University, Guangzhou 510632, China.; 3 Guangdong Provincial Key Laboratory of Bioengineering Medicine, National Engineering Research Center of Genetic Medicine, Jinan University, Guangzhou 510632, China.; 4 Department of Laboratory Medicine, Third Affiliated Hospital of Sun Yat-sen University, Guangzhou 510630, China.

**Keywords:** Mesenchymal stem cells, Extracellular vesicles, miR-206-3p antagomir, Alzheimer's disease

## Abstract

**Rationale:** The level of miR-206-3p in the plasma and temporal cortex is increased in Alzheimer's disease (AD) patients. miR-206-3p antagomir injected into hippocampus ameliorates cognitive deficits by enhancing the level of BDNF. However, the trauma caused by brain injection and susceptibility to degradation limit its application.

**Methods:** To overcome these challenges, we constructed engineered extracellular vesicles derived from mesenchymal stem cell (MSC-EVs) loaded with miR-206-3p antagomir (MSC-EVs-anta) by electroporation technology, and explored the therapeutic effects of MSC-EVs-anta delivered by intranasal administration on AD mice. Transcriptome sequencing and LC-MS/MS proteomic analysis were employed to disclose the mechanism underlying the attenuation of AD phenotypes by MSC-EVs-anta.

**Results:** MSC-EVs-anta had favorable neuroprotection by promoting neurite outgrowth *in vitro*. Following intranasal administration, MSC-EVs-anta improved learning and memory deficits, promoted hippocampal neurogenesis and synaptic plasticity, and alleviated Aβ deposition. Compared with MSC-EVs or miR-206-3p antagomir alone, MSC-EVs-anta showed superior therapeutic effects. Mechanistically, MSC-EVs-anta significantly upregulated brain-derived neurotrophic factor (BDNF) in AD mice, and activated the BDNF/TrkB signaling pathway. The data from two-omics analyses demonstrated that the differentially expressed proteins and genes significantly regulated by MSC-EVs-anta were primarily enriched in the pathways involved in neurogenesis and synapse.

**Conclusions:** Our findings highlight the intranasal administration of MSC-EVs-anta as a promising strategy for the treatment of AD.

## Introduction

Alzheimer's disease (AD) is the most common neurodegenerative disorder, accounting for approximately 80% of dementia cases. It is characterized by deposition of senile plaques, neurofibrillary tangles, along with associated loss of synapses and neurons, chronic inflammation, which results in cognitive decline 1. The number of AD patients worldwide is close to 50 million 2, with approximately 10 million in China 3. Early AD therapy relies on acetyl-cholinesterase inhibitors (donepezil, galantamine, and rivastigmine) and N-methyl-D-aspartate (NMDA) receptor antagonist (memantine) 4, 5. In the last 3 to 5 years, two approved AD therapeutic drugs (sodium oligomannate and aducanumab) have shown efficacy in attenuating AD symptoms, but the therapeutic effect remains controversial 6, 7. Recently, FDA has accelerated the approval of Lecanemab that reduces the deposition of Aβ in the brain and alleviates memory decline 8. However, longer-term trials are needed to ensure its safety and effectiveness in early AD. Therefore, new AD treatment strategies are still imperative.

Preclinical evidences suggest that mesenchymal stem cells (MSCs) have enormous potential in the treatment of neurological disorders, mainly due to their immunomodulation and tissue regeneration 9, 10. The anti-inflammatory and neuroprotection potential of MSCs have been confirmed in the treatment of ischemic stroke 11, traumatic brain injury 12, Parkinson's disease 13 and AD 14. However, MSC-based therapy is limited by the loss of cellular homeostasis and stem cell function, immune response, and ethical issues 15-17. Extracellular vesicles (EVs) are membrane-bound vesicles with diameters of 30-150 nm that participate in cellular communication. MSC-derived EVs (MSC-EVs) have more advantages in therapeutic potential than MSCs, including low immunogenicity, high stability, no tumorigenicity and easy passing through blood-brain barrier (BBB) 18. MSC-EVs have been demonstrated to ameliorate cognitive impairments in AD mice by reducing Aβ accumulation 19, relieving neuronal damage 20, and alleviating inflammation and oxidative stress 21, 22. Although MSC-EVs represent an ideal potential therapy for AD 19, its application is still hindered by nonuniform treatment outcomes and low efficiency 23. Therefore, engineering techniques help to improve the therapeutic efficacy of MSC-EVs, which is of great significance for the treatment of AD.

Engineered EVs have shown potential in the treatment of various diseases in recent years 24, 25. Therapeutic molecules, such as nucleic acids, peptides and proteins, can be loaded into native EVs by endogenous and exogenous loading approaches. miR-29b-loaded EVs derived from bone marrow MSCs transfected with miR-29b contributed to improve memory deficit in AD mice 26. EVs secreted by adipose-derived MSC transfected with anti-miR-25-3p oligonucleotides showed neuroprotective effects on stroke mice 27. Endogenous methods are used to construct engineered MSC-EVs by transfecting source cells with vectors. However, the potential risks associated with the exogenous factors (e.g., transfection regents and lentiviral vectors), uneven cargo levels and transfection efficiency restrict their application. In comparison, exogenous approaches enable EVs to load specific drugs, which improve control over cargo loading. As a common physical approach, loading efficiency of electroporation is higher than that of chemical transfection 28, sonication, incubation, extrusion, saponin permeabilization or hypotonic dialysis 29, 30. Electroporation technology makes specific drug loading feasible, and optimizing the electroporation protocol is crucial for efficient cargo loading.

We have found that AD individuals showed high miR-206-3p levels in the plasma, and reducing miR-206-3p expression through brain injection with antagomir can upregulate the level of brain-derived neurotrophic factor (BDNF), decrease Aβ plaque, and enhance learning and memory abilities in AD mice 31. Intracerebral injection is highly invasive and not suitable for long-term treatment, whereas intranasal delivery is non-invasive for the administration of miR-206-3p antagomir. However, it still faces the limitation of low absorption rates and susceptibility to degradation. For these reasons, we sought to construct engineered MSC-EVs loaded with miR-206-3p antagomir (MSC-EVs-anta) by electroporation technology, and explore the therapeutic effects of MSC-EVs-anta delivered by intranasal administration on AD pathology and behavior. After intranasal delivery, we assessed the ability of MSC-EVs-anta to enter the brain, ameliorate cognitive behavior decline, reduce Aβ burden and promote neurogenesis. Subsequently, transcriptome sequencing and LC-MS/MS proteomic analysis were employed to disclose the mechanism underlying the attenuation of AD phenotypes by MSC-EVs-anta. Our study will contribute to the development of a new AD treatment strategy.

## Materials and Methods

### Animal models and treatment

8-week-old male C57BL/6J mice (24-28 g) were purchased from the animal research center of Changsheng Bio-technology Co. Two weeks before treatment, all animals were acclimatized under the specific-pathogen-free environment of suitable humidity (50%-60%) and temperature (24 ± 1 ℃) with a 12 h-light/dark cycle. All animal experiment processes were performed following the Chinese Institutional Guidelines for Animal Care and Use and approved by the animal ethics committee of Jinan University (Approval number: IACUC-20191011-01).

An AD animal model was induced by slowly injecting 4 μL of Aβ_1-42_ oligomer (2 μg/μL, 4 μg/side and 8 μg/mouse, Qiangyao Biotechnology, Shanghai, China) into the bilateral hippocampus (bregma, ± 1.5 mm lateral, -2.3 mm posterior, 2.36 mm deep) of mice as described previously 31. Aβ_1-42_ oligomers was administered as a continuous infusion at a rate of 0.8 μL/min using a microinjection pump (RWD life science, Shenzhen, China). After three weeks of brain injection, the AD mice were randomly divided into six groups: sham, Aβ, Aβ+MSC-EVs, Aβ+MSC-EVs-nc, Aβ+MSC-EVs-anta and Aβ+anta. After treated with Aβ_1-42_ oligomer for three weeks, mice were intranasally administered with MSC-EVs, MSC-EVs-nc, MSC-EVs-anta, miR-206-3p antagomir and volume-matched phosphate buffered saline (PBS) respectively, every 2 days for 6 weeks according to the previous protocol 31. In brief, the nasal mucous membrane was permeabilized with 3 μL hyaluronidase (100 U/mouse, S10060, Shanghai Yuanye Bio-Technology Co., Ltd) for 30 minutes. Subsequently, each nostril received a total volume of ~8 μL of MSC-EVs (5.22×10^8^), MSC-EVs-nc (MSC-EVs loaded with antagomir NC), MSC-EVs-anta (MSC-EVs loaded with ~10 pmol miR-206-3p antagomir), miR-206-3p antagomir (~10 pmol) or PBS by a 10-μL micropipette (~2.5 μL each time, lasted for 3 minutes).

### Cell culture and isolation of MSC-EVs

Bone marrow mesenchymal stem cells (MSCs) were isolated from rat using the previously reported method 32. Briefly, 4-week-old Sprague-Dawley (SD) were euthanized to separate the femurs. Bone marrow cavity was rinsed with Dulbecco's Modified Eagle's Medium (DMEM; Gibico, USA) using an 18-gauge needle. After filtering through a 70 μm mesh to remove debris, the suspension was centrifuged at 300 g for 5 minutes. Bone marrow cells were seeded in 75 cm^2^ culture flasks in DMEM supplemented with 10% fetal bovine serum (FBS, Natocor, Argentina). After a 12-hour incubation, non-adherent cells were removed and culture medium was replaced every 3 days. When cells were passaged to the 3nd generation, the purity of the isolated MSCs was determined by immunofluorescence staining with MSC-specific positive markers CD90 (1:100, Rabbit, DF4804, Affinity, USA) and CD73 (1:100, Rabbit, DF6763, Affinity, USA), as well as negative marker CD45 (1:100, Rabbit, DF6839, Affinity, USA). The 4th to 6th generation cells were used in the experiments. To obtain EVs derived from MSCs, MSCs were incubated with DMEM supplemented with 2% exosome-free FBS (EXO-FBS-50A-1, SBI, USA) for 72 hours. The MSC-EVs were isolated from the culture supernatant by ultracentrifugation as previously described 33. Firstly, the freshly harvested culture supernatant was centrifuged at 300 g for 10 minutes, 1,000 g for 10 minutes, and 10,000 g for 30 minutes at 4 ℃ to remove cellular debris. Then, the supernatant was ultracentrifuged at 100,000 g for 2 hours at 4 ℃ (CP70ME, Hitachi, Japan). Lastly, the pellet was washed with PBS and ultracentrifuged at 100,000 g for 2 hours at 4 ℃. The purified MSC-EVs were characterized by immunoblotting, nanoparticle tracking analysis (NTA), and transmission electron microscope analysis 33. The expression of EV specific marker CD9 (1:1000, Rabbit, DF6565, Affinity, USA), CD63 (1:1000, Rabbit, AF5117, Affinity, USA), CD81 (1:1000, Rabbit, DF2306, Affinity, USA) and TSG101 (1:1000, Rabbit, DF8427, Affinity, USA) were detected by immunoblotting, with Calnexin (endoplasmic reticulum-specific biomarker, 1:000, Rabbit, abs131462, Absin, China) being a negative marker. The concentration and size distribution of MSC-EVs were measured by Flow Nanoanalyzer (NTA, NanoFCM N30E). The structure of EVs were detected by transmission electron microscope analysis (HT7700, Hitachi High Technologies, Tokyo, Japan).

Primary cortical neurons and hippocampal cells were isolated from the brains of 1 to 3-day-old SD rats as described previously 34. The brain tissues were minced and trypsinized in 0.125% trypsin-EDTA. DMEM supplemented with 10% FBS was added to terminate the digestion after 10 minutes. Tissue blocks were gently dispersed using a glass pipette, and the supernatant was then passed through a 200-mesh filter to remove clumps. Subsequently, the cell suspension was centrifuged at 1,000 rpm for 5 minutes. For primary cortical neurons, cells were seeded on the culture plate in DMEM supplemented with 10% FBS. After 6 hours of cultivation, the medium was replaced with the neurobasal medium (21103049, Life, USA) supplemented with 2% B27 (50 ×, 17504044, Invitrogen, USA). The culture medium was changed every 3 days, and the primary cortical neurons were cultured for 5 days before experiments. For primary hippocampal cells, the filtered cells were cultured in neurobasal medium supplemented with 10 % FBS for 7 days, with the culture medium changed every 3 days.

### MSC-EVs loading experiments

The engineered MSC-EVs were constructed by electroporation performed on a multi-electroporator (Eppendorf International, Germany) according to the manufacturer's instructions 35. Briefly, MSC-EVs (6.53×10^8^ particles) and miR-206-3p antagomir (10 or 20 pmol) were mixed and incubated for 30 minutes at 4 °C. The EVs-miR-206-3p antagomir mixture was added to 200 μL electroporation buffer in electroporation cuvette (0.2 cm), and subjected to an electroporation using a pulse width of 20 ms and different voltages (750 V, 1000 V, and 1250 V). Subsequently, the mixture was incubated at 37 °C for 30 minutes and overnight at 4 °C. MSC-EVs and miR-206-3p antagonist were incubated without electroporation, which was used as a control along with electroporated MSC-EVs. To remove the unbound miR-206-3p antagomir, the mixed samples were treated with RNase A (at a concentration of 0.2 μg/mL) at 37 °C for 30 minutes. After terminating by RNase inhibitor (Thermo Fisher Scientific), the engineered MSC-EVs were washed and separated by 100,000 g for 2 hours at 4 ℃. The engineered MSC-EVs (MSC-EVs-anta) were resuspended in PBS and stored at -80 °C for subsequent experiments. Loading efficiency was calculated using the formula: miR-206-3p antagomir loading efficiency (%) = (Amount of loaded miR-206-3p antagomir/Total amount of miR-206-3p antagomir used) × 100 36. Based on the reported study 35, we evaluated the total RNA content in MSC-EVs as a visual indicator for assessing loading efficiency.

### Characterization of MSC-EVs-anta

To quantify the loaded RNA in engineered MSC-EVs, total RNA was extracted from MSC-EVs-anta using Trizol reagent (Life Technologies). The control groups included incubation of MSC-EVs with miR-206-3p antagomir simply, and electroporated MSC-EVs without miR-206-3p antagomir. The concentration and purity of total RNA were measured using spectrophotometer (NanoDrop 2000, Thermo Fisher Scientific). In order to analyze the influence of electroporation on MSC-EVs, the particle size, shape and markers of EVs were detected by nanoparticle flow cytometer, Transmission electron microscopy (TEM) and immunoblot analysis respectively.

### Histopathology of brain

Male mice were euthanized after intranasal administration of ~16 μL MSC-EVs (1.04×10^9^) or volume-matched PBS for 6 weeks. The brain tissues were isolated and fixed in 4% paraformaldehyde. The brain sections stained with Hematoxylin-Eosin (H&E) were visualized and imaged using a light microscope (Nikon, Japan).

### Tracing experiment of MSC-EVs *in vivo* and *in vitro*

To track MSC-EVs in the brain, 60 μg of engineered MSC-EVs stained with PKH26 (Sigma-Aldrich, MIDI26, USA), and an equal volume of PBS containing the PHK26-stained centrifuged precipitates were intranasally administrated to AD mice. After 6 hours, the brains were isolated and fixed in 4% paraformaldehyde. The brain sections (10 μm) were incubated with the following primary antibodies to track the distribution of EVs in the brain: rabbit anti-NeuN (neuron marker, Abcam, 1:300, ab209898), rabbit anti-Iba1 (microglia marker, Abcam, 1:300, ab283319) and rat anti-GFAP (astrocyte marker, CST, 1:300, 3670S).* In vitro*, the uptake of engineered MSC-EVs was tracked by incubating neurons with 5 μg MSC-EVs (PKH26-labeled, red) loaded with FAM-labeled miR-206-3p antagomir (~2 pmol, green) for 0.5, 4 and 24 hours, respectively. Subsequently, neurons under bright-field, the labeled MSC-EVs and miR-206-3p antagomir were visualized under laser scanning confocal microscopy (LSM 900, Carl Zeiss, Germany). The co-localization of miR-206-3p antagomir with MSC-EVs in neurons was analyzed using Pearson's coefficient.

### Cellular and tissue immunofluorescence

Cells were fixed with 4% paraformaldehyde for 15 minutes and permeabilized with 0.2% tritonX-100 (Sigma) for 20 minutes. The samples were blocked with 5% bovine serum albumin (BSA) for 1 hour at room temperature, and sequentially incubated with rabbit monoclonal anti-β-III-tubulin antibody (Abcam, 1:200, ab11267) and Alexa flour® 488 goat anti-rabbit IgG (Life Technologies, 1:300, A11008). Then nuclei were stained with DAPI for 10 minutes. The images were captured by confocal microscope (LSM 900, Carl Zeiss, Germany), and all images were analyzed by image-pro-plus software.

Paraffin-embedded brain tissues were deparaffinized, rehydrated, and treated with 3% H_2_O_2_ solution for 10 minutes. The tissue specimens were incubated in 0.01 M citrate buffer solution for antigen retrieval and blocked with 5% BSA at room temperature for 1 hour. Next, the specimens were incubated with mouse anti-6E10 antibody (Biolegend, 1:200, 803014), rabbit anti-NeuN antibody (Abcam, 1:200, ab177487) and rabbit anti-Dcx antibody (Abcam, 1:200, ab18723) overnight at 4 °C respectively. On day 2, the specimens were incubated with diluted Alexa flour^®^ 594 goat anti-mouse IgG (Life Technologies, 1:300, A11005) or Alexa flour® 488 goat anti-rabbit IgG (Life Technologies, 1:300, A11008) at room temperature for 1 hour, followed by staining with DAPI for 10 minutes. The images of slices were obtained by confocal microscope and analyzed by Image-Pro Plus software.

### Western blotting and dot blotting

The tissues and cells were sonicated in RIPA lysis buffer containing protease and phosphatase inhibitors, and the supernatants were obtained after centrifugation at 12,000 rpm for 30 minutes at 4 °C. The protein concentration was determined using the BCA protein assay kit (ThermoFisher Scientific). A total amount of 30 μg protein was loaded on 12% or 15% SDS-PAGE gel, transferred to polyvinylidene difluoride (PVDF) membranes (Millopore, Billerica, USA), and detected by Western blotting with antibodies against PSD95 (CST, 1:1000, D74D3), SYN (Abcam, 1:1000, ab32127), GAP43 (Abcam, 1:1000, ab75810), BDNF (Abcam, 1:1000, ab108319), 4G8 (Biolegend, 1:1000, SIG-39220), TrkB (Affinity, 1:1000, AF6461), p-TrkB (Affinity, 1:1000, AF3461), PLCγ1 (Affinity, 1:1000, AF6210), p-PLCγ1 (Affinity, 1:1000, AF3210), Akt (CST, 1:1000, 9272S), p-Akt (Ser473) (CST, 1:1000, 4060T), ERK (CST, 1:1000, 4695T), (CST, 1:1000,4370T), CREB (CST, 1:1000, 4820S), p-CREB (CST, 1:1000, 9198T), β-III-tubulin (Abcam, 1:5000, ab11267), β-actin (1:5000, Bioworld, BS6007M), and GAPDH (CST, 1:1000, 5174T) respectively. The membranes were incubated with primary antibodies overnight at 4 °C, and incubated with secondary antibodies (FD Biotechnology, 1:5000) for 1 hour at room temperature. The hippocampal samples were also detected by dot blotting, and were labled by rabbit anti-oligomer A11 antibody (Invitrogen, 1:1000, AHB0052). Signals were visualized using ECL kit (Thermo Fisher Scientific, USA). The intensity of the bands was analyzed using ImageJ software.

### Golgi staining

The Golgi staining was performed following the instructions of modified Golgi-Cox kit (GMS80020.1). The cerebral hemispheres of mice were fixed in the impregnation solution in dark for 2 weeks. Subsequently, the brain tissue was dehydrated in a 30% sucrose solution for 48 hours, then were coronally sectioned into tissue pieces with a thickness of 100 μm. The pieces were subjected to deparaffinization and hydration, and stained for 30 minutes at room temperature. The slices were dyed in ammonia water for 15 minutes, sealed with neutral resin.

### *In situ* hybridization of miR-206-3p

The right brain hemispheres of AD mice were sectioned coronally with a thickness of 6-μm. The sections were sequentially treated with hydrochloric acid, 0.5% tritonX-100 and proteinase K (20 μg/mL) for 12 minutes at room temperature, and then hybridized with digoxigenin-labeled probes (1:100, Focibio) at 41 ℃ for 48 hours. After washing with PBS, the sections were incubated with anti-digoxigenin HRP (1:100, Focobio) at 37 ℃ for 1 hour, followed by a 15-minute incubation with TSA (green, Focobio). Images were captured by a confocal microscope, and processed with ImageJ software.

### Morris water maze

Learning and memory abilities of AD mice were assessed by the Morris water maze (MWM) test as described previously 31. The maze apparatus includes a circular pool (120 cm diameter, 50 cm height), a submersible platform (8 cm diameter), video recorder and a computer camera analysis system. For the navigation test, a submersible platform (1 cm below the water level) was placed for mice to escape. Each mouse was trained with two trials (once per quadrant) per day (interval between each trial over 20 minutes). During training trials, the mouse was gently placed into the water, facing the wall of the pool. The time and movement tracks of each mouse then were recorded in 60 seconds. If the mouse found the platform and stayed on it for 4 seconds, the recorder automatically stopped; otherwise, the mouse was guided to the platform and stayed on it for 10 seconds. After a 24-hour rest period following the last train, the spatial probe test was conducted. The platform was removed, and the times of crossing the removed-platform area and time spent in the target quadrant of the platform were recorded within 90 seconds.

### RNA isolation and RT-PCR/qPCR

Total RNA or miRNA was extracted using the Trizol reagent (Invitrogen, 15596026CN). cDNA was synthesized with the reverse transcription reagent kit (Takara, RR037) or the all-in-one miRNA First-Strand cDNA synthesis kit (GeneCopoeia, QP013). For mRNA analysis, qPCR or RT-PCR was performed using a PCR system containing 2 μL cDNA, 7.2 μL free-RNase H_2_O, 0.8 μL forward and reverse primers, and 10 μL SYBR Green PCR master mixes or PCR master mixes. For miR-206-3p detection, cDNAs were amplified according to the instructions of the All-in-One miRNA RT-qPCR Detection Kit (GeneCopoeia, QP016). The primers synthesized by Tianyi Huiyuan (Guangzhou, China) or GeneCopoeia (Guangzhou, China) are shown in Table 1.

### Transcriptome sequencing

Total RNA was isolated from hippocampal tissues of mice. The purity and quantification of RNA were assessed using the NanoDrop 2000 spectrophotometer (ThermoScientific, USA), and the integrity of RNA was evaluated using the Agilent 2100 Bioanalyzer (Agilent Technologies, Santa Clara, CA, USA).

The transcriptome library was established using the VAHTS Universal V5 RNA-seq Library Prep kit according to the instructions, and then sequenced using the Illumina Novaseq 6000 platform. After aligning the readings to a reference genome, gene reading counts were obtained. The DESeq2 software was utilized to normalize counts and analyze the differential expression of genes. Genes with fold change ≥ 2 or ≤ 0.5, and q-value < 0.05 were defined as differentially expressed genes (DEGs). Enrichment analyses of DEGs, including GO, KEGG Pathway, GSEA and WikiPathways, were conducted using the hypergeometric distribution algorithm. The transcriptome sequencing and data analysis were performed by Ouyi Biotechnology Co., Ltd. (Shanghai, China).

### LC-MS/MS proteomic analysis

Total protein was extracted from mouse hippocampal tissues. Followed by concentration determination and SDS-PAGE quality testing, proteins were subjected to trypsin digestion and TMT labeling. Subsequently, equal amounts of each labeled samples were mixed, chromatographically separation, and analyzed by LC-MS/MS. Raw data analysis was processed using Proteome Discoverer 2.4.1.15 (ThermoFisher Scientific, USA) with reference to the uniprot fasta database (uniprot-Mus musculus-10090-2023.2.1). Fold change ≥ 1.2 or ≤ 1.2, and *p*-value < 0.05 were set as significant level. Enrichment analysis of proteins was performed using KEGG pathway, Gene ontology (GO), Reactome pathway, WikiPathways and Gene Set Enrichment Analysis (GSEA).

### Statistical analysis

GraphPad prism (version 8.2.1, Graphpad Software, Inc) was applied for statistical analysis and graphing of the data. The D'Agostino-Pearson test was used to determine the normality of data, and the data was confirmed to be normally distributed when *P* > 0.05. Subsequently, one-way analysis of variance (One-way ANOVA) with Tukey's *post-hoc* multiple comparison was used to analyze and compare the differences among three or more groups. Two-group comparisons were analyzed by Student's *t*-test. Data were presented as mean ± standard error of mean (Mean ± SEM). *P* < 0.05 was considered statistically significance. Western blot bands were quantified for grayscale intensity using ImageJ software, and neurite length and number were calculated using Image Pro-plus software.

## Results

### Characteristics of MSC-derived extracellular vesicles loading miR-206-3p antagomir

**Figure 1:**
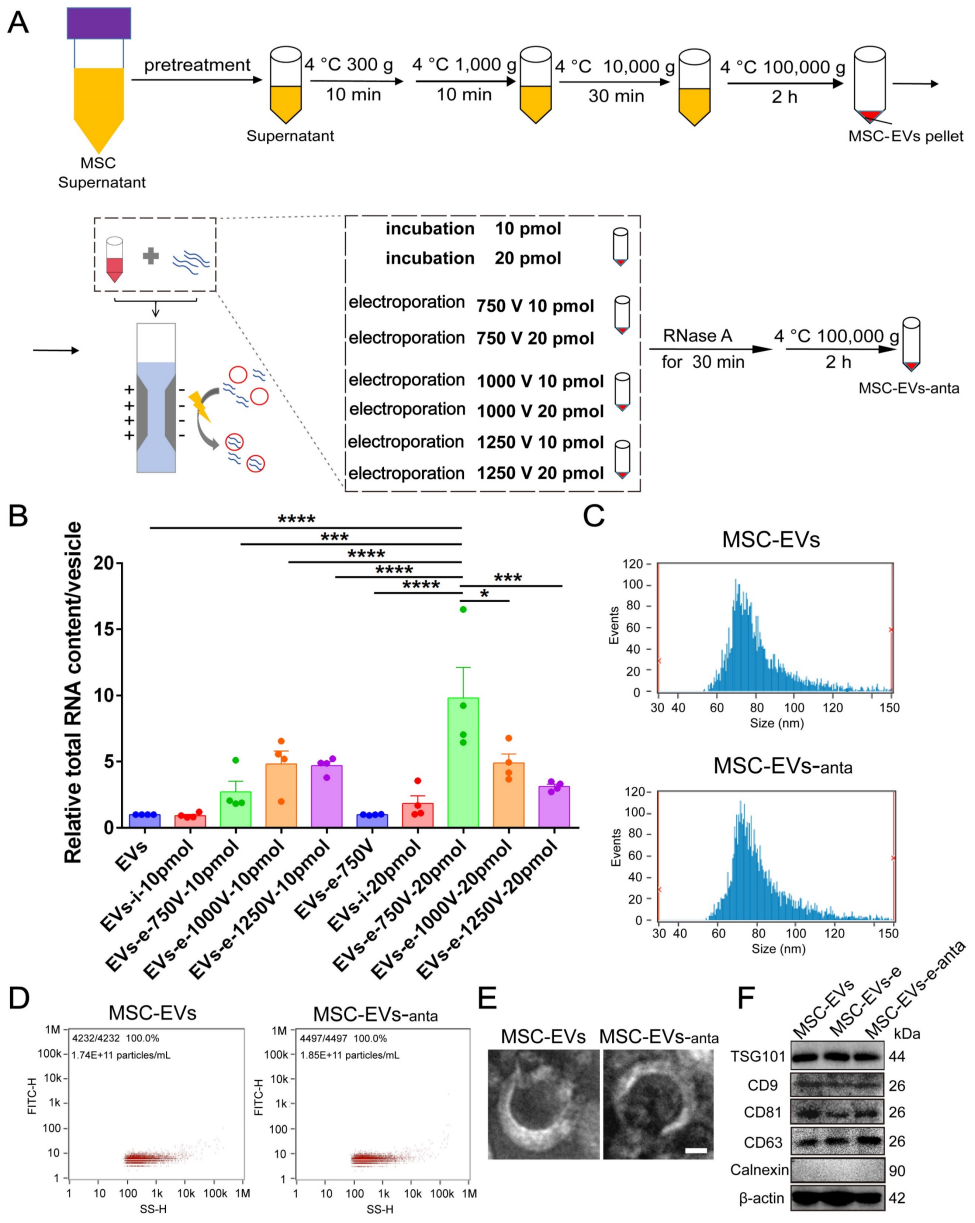
** Preparation and characterization of MSC-derived extracellular vesicles loaded with miR-206-3p antagomir. (A)** Schema illustrates the process of constructing engineered MSC-EVs through electroporation. **(B)** Total RNA was extracted from engineered MSC-EVs and quantified. The RNA content in MSC-EVs was evaluated under different treatment conditions, n = 4.** (C-E)** Representative size distribution **(C-D)** and TEM images **(E)** of MSC-EVs and MSC-EVs-anta. **(F)** The expression of CD9, CD63, CD81, TSG101, β-actin and negative marker Calnexin in naive MSC-EVs, electroporated MSC-EVs, and MSC-EVs-anta was determined by immunoblotting. The data is presented as mean ± SEM. *P < 0.05, ***P < 0.001, ****P < 0.0001. P values are calculated by one-way ANOVA with Tukey's *post-hoc* test.

As a vehicle for miR-206-3p antagomir, EVs were isolated from the cultured medium of bone-marrow-derived MSCs according to the previous protocol 35 (Figure 1A and S1). To optimize the loading capacity of EVs, different voltages (750, 1000 and 1250 V) and different miR-206-3p antagomir contents (10 and 20 pmol) were applied during the electroporation process. The capacity of miR-206-3p antagomir loading was evaluated by the total RNA content in MSC-EVs. The RNA contents were increased when miR-206-3p antagomir was transfected into MSC-EVs by electroporation. The setting with 750 V and the amount of miR-206-3p antagomir at 20 pmol allowed for maximum RNA enrichment (Figure 1B). The optimal loading efficiency of miR-206-3p antagomir in MSC-EVs was calculated to be 46.9% using the above formula. The particle size displayed by NanoSight (NTA) had no differences between electroporated MSC-EVs and control MSC-EVs (Figure 1C-D). TEM imaging showed that both the electroporated MSC-EVs and control MSC-EVs exhibited bilipid-layered and nano-sized particles (Figure 1E). The expression of CD9, CD63, CD81, TSG101 and β-actin were both positive in MSC-EVs and MSC-EVs-anta, while calnexin was negative (Figure 1F). These results suggest that electroporation has no influence on the particle size, shape, and markers of MSC-EVs, and MSC-EVs loaded miR-206-3p antagomir (MSC-EVs-anta) are successfully generated.

### MSC-EVs-anta promote neurite outgrowth *in vitro*

**Figure 2:**
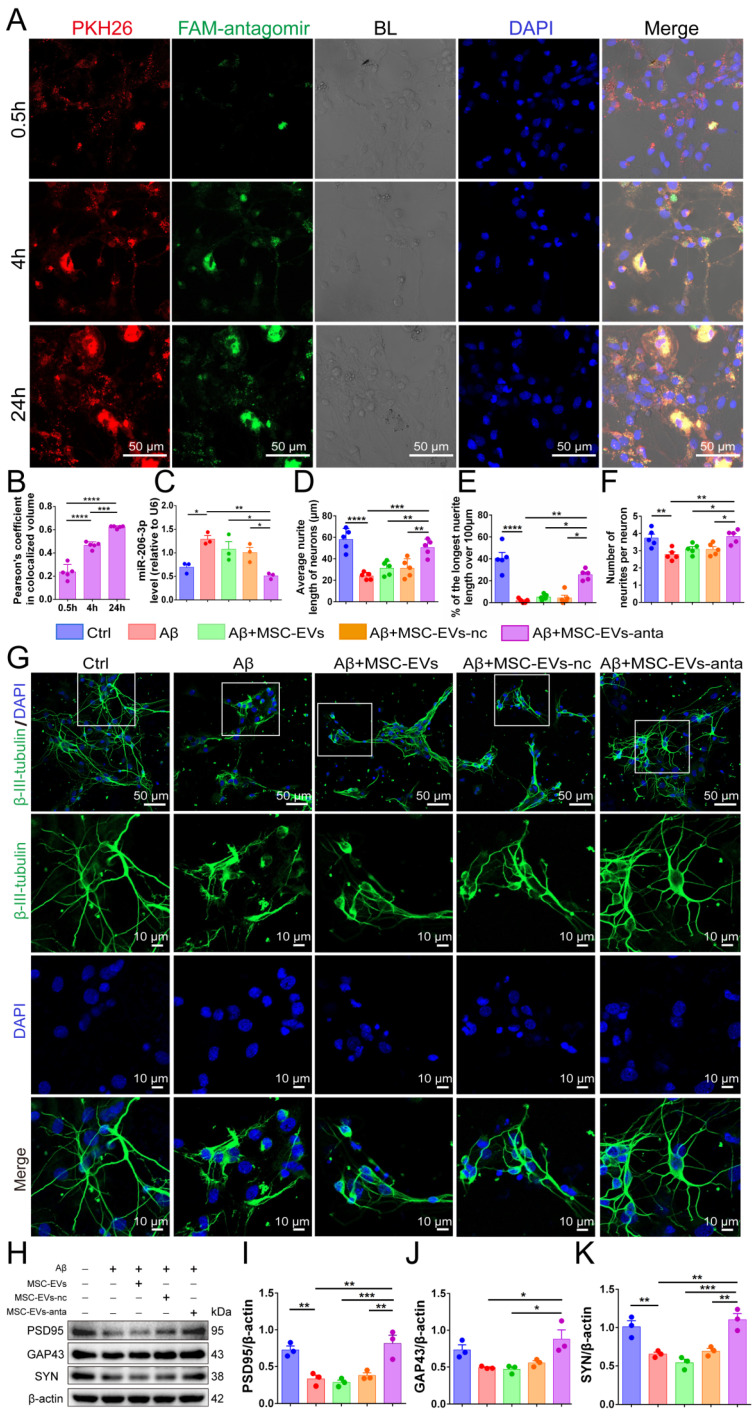
** MSC-EVs-anta promote neurite outgrowth in neurons *in vitro.* (A)** Representative images of MSC-EVs-anta (loaded with FAM-miR-206-3p antagomir) labeled with PKH26 (red) in primary cortical neurons (Scale bars: 50 μm). Neurons and MSC-EVs-anta were co-culture for 0.5, 4 h, and 24 hours. **(B)** Co-localization analysis of MSC-EVs and FAM-miR-206-3p antagomir was assessed by Pearson correlation coefficient, n = 5.** (C)** The levels of miR-206-3p detected by qPCR in primary cortical neurons damaged by Aβ after co-culture with MSC-EVs-anta, n = 3. **(D-G)** The β-III-tubulin^+^ cells (green) in rat primary cortical neurons treated with MSC-EVs-anta under the condition of Aβ-damage.** (G)** The representative images of neurons (Scale bars: 50 or 10 μm). The average neurite length of neurons **(D)**, the proportion of the longest length of neural neurites over 100 µm** (E)** and the number of neural neurites per neuron** (F)** were evaluated by image-pro plus, n = 5. **(H)** The expressions of PSD95, GAP43, SYN and β-actin were determined by Western blotting. **(I-K)** The levels of PSD95, GAP43 and SYN were quantified by normalization to β-actin, n = 4. The data is presented as mean ± SEM. *P < 0.05, **P < 0.01, ***P < 0.001, ****P < 0.0001. P values are calculated by one-way ANOVA with Tukey's *post-hoc* test.

MSC-EVs loaded with FAM-tagged miR-206-3p antagomir were added to the culture medium of primary cortical neurons (DIV 5). Following incubation for 0.5, 4 and 24 hours, PKH26-labeled MSC-EVs (red) and FAM-labelled miR-206-3p antagomir (green) were distributed in the cytoplasm of neurons, and the uptake increased over time (Figure 2A-B). However, when incubated with free miR-206-3p antagomir, its uptake in neurons was very low. The uptake of miR-206-3p antagomir in neurons damaged by Aβ_1-42_ was higher than that in healthy neurons, and the uptake has no difference in different time points (Figure S2A-B). Furthermore, we found that MSC-EVs-anta significantly reduced the level of miR-206-3p in neurons damaged by Aβ_1-42_, whereas this effect was not observed in MSC-EVs or MSC-EVs-nc treatments (Figure 2C).

Next, primary cortical neurons were subjected to the damage induced by Aβ_1-42_ oligomer to establish an *in vitro* AD model. Aβ_1-42_ induced neuronal damage, as evidenced by the reduced average neurite length, decreased neurite number and percentage of the longest neurite length (> 100 μm). Compared with Aβ_1-42_ and MSC-EVs treatment, MSC-EVs-anta obviously promoted the three parameters associated with neurite outgrowth. MSC-EVs alone had no significantly improvement on Aβ_1-42_-damaged neurons, which might be possibly related to the low dose of MSC-EVs in treatment (Figure 2D-G). Aβ_1-42_ also inhibited the expression of postsynaptic density protein 95 (PSD95, markers of postsynaptic integrity) and synaptic plasticity-related proteins (SYN, markers of presynaptic integrity), but did not inhibit the expression of growth-associated protein 43 (GAP43, a marker of axonal sprouting). Compared with Aβ_1-42_ and MSC-EVs treatment, PSD95, SYN, and GAP43 expression levels in neurons were remarkably enhanced after incubating with MSC-EVs-anta (Figure 2H-K). These results demonstrate that MSC-EVs-anta have neuroprotection by promoting neurite outgrowth.

### MSC-EVs-anta reduce Aβ burden in the brain and ameliorate AD cognitive decline

To track the distribution of MSC-EVs in the brain, mice were treated intranasally with PKH26-labeled MSC-EVs. After 6 hours intranasal administration of PKH26-labeled MSC-EVs, strong fluorescence was detectable in the prefrontal cortex (PFC) and hippocampus (Figure S3A). To further investigate the cell types that uptake MSC-EVs in the brain, the brain tissues were stained by anti-Neu (neuron), anti-GFAP (astrocyte) and anti-Iba1 (microglia) signals (Figure S3A). The results showed that MSC-EVs were mainly distributed in neurons, and microglia took up more MSC-EVs than astrocytes.

**Figure 3:**
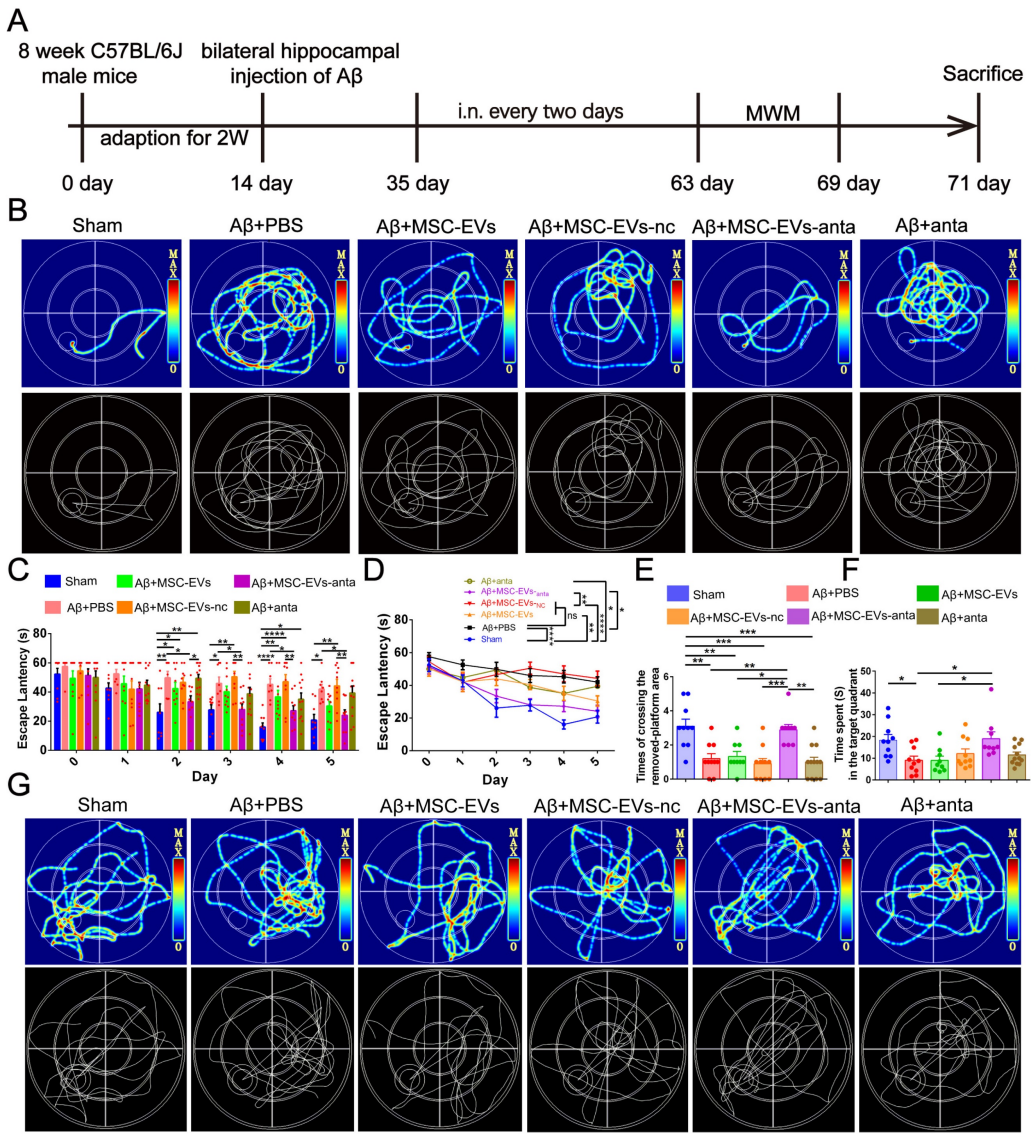
** MSC-EVs-anta ameliorate cognitive deficits in AD mice. (A)** The experimental schedule for intranasal administration and behavioral testing in AD mice. **(B)** The representative movement tracks of escape latency (day 5) in navigation test of MWM.** (C-D)** The escape latency of MWM, n = 8-11.** (E-G)** Representative path patterns in spatial probe test of MWM** (G)**, and the times of crossing the removed platform area **(E)** and time spent in target quadrant** (F)** were recorded and analyzed, n = 8-11. The data is presented as mean ± SEM. *P < 0.05, **P < 0.01, ***P < 0.001, ****P < 0.0001. P values are calculated by one-way ANOVA with Tukey's post-hoc test or two-way ANOVA with Tukey's *post-hoc* test.

To assess the improvement of MSC-EVs-anta in cognitive deficient, the Morris water maze (MWM) test was conducted, following intranasal administration of MSC-EVs-anta to AD mice every 2 days for 6 weeks (Figure 3A). On the 5^th^ day of escape latency testing, the representative movement tracks of mice are shown in Figure 3B. From day 2 to day 5, the mice intranasally receiving MSC-EVs-anta displayed a significant reduction in escape latency compared with AD mice treated with PBS, MSC-EVs or miR-206-3p antagomir (Figure 3C-D). In the space probe test, AD mice treated with MSC-EVs-anta performed a dramatic improvement, as evidenced by the times of crossing over the removed-platform area and time spent in the target quadrant (Figure 3E-G).

**Figure 4:**
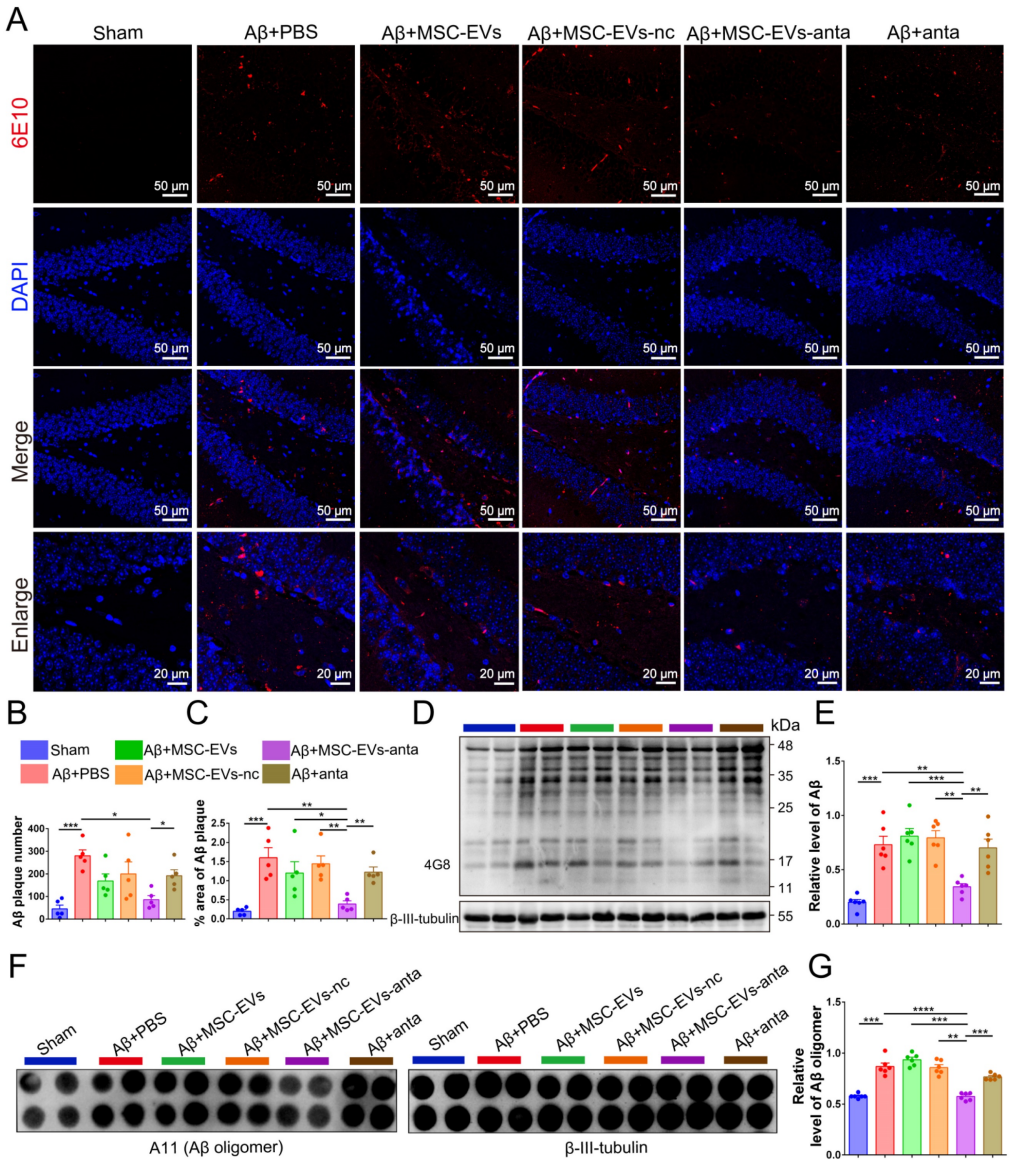
** MSC-EVs-anta reduce Aβ burden in AD brain. (A)** Aβ plaque in the dentate gyrus (DG) regions of hippocampus was stained by immunofluorescence. **(B-C)** The number and area of Aβ plaque are analyzed by image-pro plus software, n = 6.** (D)** The representative immunoblots of Aβ in the hippocampus, and **(E)** quantified expression of Aβ normalized to β-III-tubulin, n = 6. **(F)** Aβ oligomers in the hippocampus were detected by dot blotting. **(G)** Relative levels of Aβ oligomers, n = 6. The data is presented as mean ± SEM. *P < 0.05, **P < 0.01, ***P < 0.001, ****P < 0.0001. P values are calculated by one-way ANOVA with Tukey's *post-hoc* test.

Since Aβ plaque deposition is the main pathological biomarkers of AD, it was detected in the hippocampus of AD mice. Immunofluorescence results showed that MSC-EVs-anta significantly reduced the number and area of Aβ plaques in the hippocampal DG region and PFC, which was superior to MSC-EVs, MSC-EVs-nc or miR-206-3p antagomir-treatment (Figure 4A-C and Figure S4A-C). Immunoblotting and dot hybridization were used to further confirm that MSC-EVs-anta alleviated Aβ burden. The Aβ oligomer level in the hippocampus and PFC of AD-mice was increased, while treatment with MSC-EVs-anta obviously resulted in a reduction of Aβ burden, but treatment with MSC-EVs, MSC-EVs-nc or miR-206-3p antagomir did not have the same effect (Figure 4D-G and Figure S4D-G).

These results indicated that MSC-EVs-anta significantly reduced Aβ deposition in the hippocampus and improved cognitive dysfunction in AD mice. MSC-EVs and miR-206-3p antagomir did not show significant improvement in cognitive impairment and Aβ burden, possibly due to the dosage and delivery method. Intranasally delivery of miR-206-3p antagomir-loaded MSC-EVs enhances the treatment effects of MSC-EVs and miR-206-3p antagomir on AD mice. It has reported that intranasal administration of MSC-EVs does not induce morphological changes in the major organs of AD mice 20. Our experimental results demonstrated that intranasal administration of MSC-EVs, MSC-EVs-nc, MSC-EVs-anta or miR-206-3p antagomir had no noticeable impacts on the brain tissue morphology (Figure S3B). MSC-EVs and miR-206-3p antagomir intranasal delivery did not result in toxicity to the brain.

### MSC-EVs-anta effectively transport miR-206-3p antagomir and activate BDNF/TrkB signaling pathway

**Figure 5:**
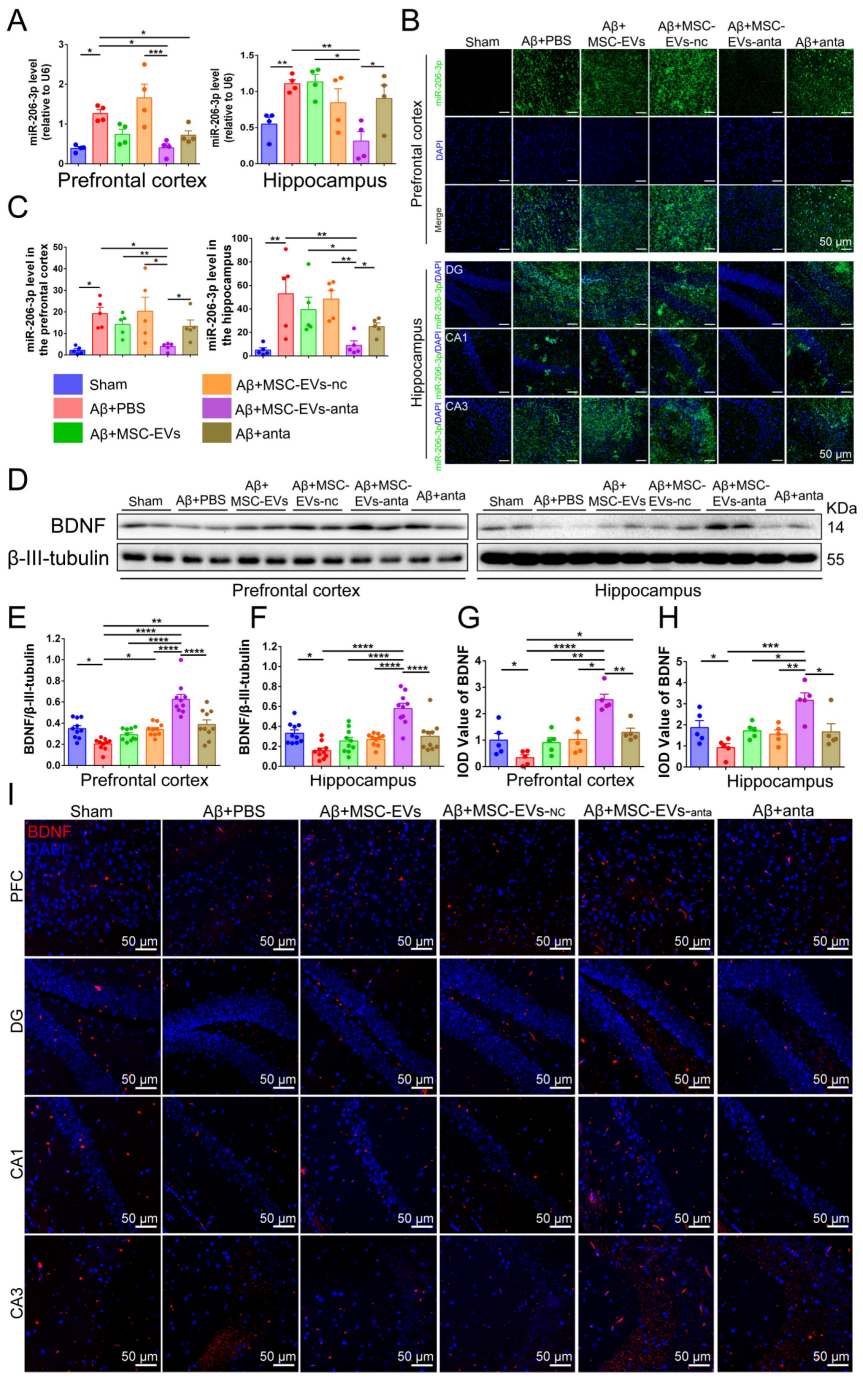
** MSC-EVs-anta transport miR-206-3p antagomir into the brain and increase BDNF levels. (A)** qPCR analyzed the expression of miR-206-3p in the PFC and hippocampus of mice after intranasal administration of MSC-EVs-anta, n = 4. **(B)** The levels of miR-206-3p in the hippocampal and PFC were measured by FISH. **(C)** Quantitative analysis of the mean fluorescence intensity of miR-206-3p, n = 5. **(D-F)** The expression of BDNF in the PFC and hippocampus of AD mice with intranasal administration of MSC-EVs, n = 10. **(G-H)** Quantitative analysis of the mean fluorescence intensity of BDNF, n = 5. **(I)** The representative images of BDNF detected by immunofluorescence in the DG, CA1, CA3 and PFC of AD mice with intranasal administration of MSC-EVs-anta. Scale bar: 50 μm. The data is presented as mean ± SEM. *P < 0.05, **P < 0.01, ***P < 0.001, ****P < 0.0001. P values are calculated by one-way ANOVA with Tukey's *post-hoc* test.

We have previously found a high miR-206-3p level in the plasma of AD individuals and the brain of AD mice, which negatively regulates BDNF transcription 31. To explore the level of miR-206-3p in the AD brain after MSC-EVs-anta administration, we tested miR-206-3p expression by qPCR and FISH. The results showed that MSC-EVs-anta significantly downregulated miR-206-3p levels in the PFC and hippocampus of AD mice (Figure 5A-C), followed by upregulation of BDNF expression (Figure 5D-I). Also, MSC-EVs, MSC-EVs-nc or miR-206-3p antagomir had a mild effect on the regulation of miR-206-3p and BDNF in AD mice.

**Figure 6:**
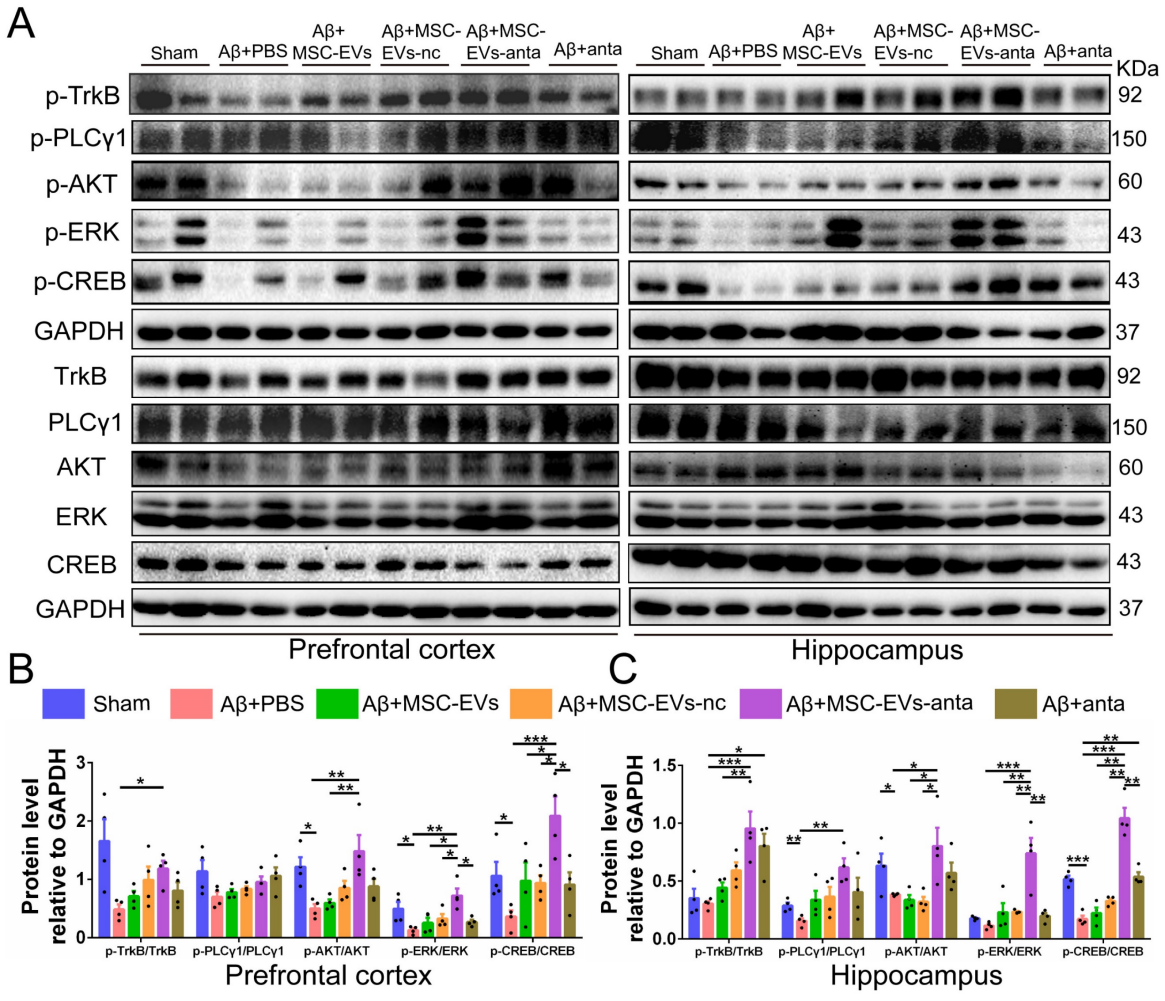
** MSC-EVs-anta activate BDNF/TrkB signaling pathway in the AD brain. (A)** The representative Western blotting images of p-TrkB, p-PLCγ1, p-Akt, p-Erk and p-CREB in the PFC and hippocampus of AD mice. **(B-K)** Quantitative analysis was performed to determine the levels of p-TrkB, p-PLCγ1, p-Akt, p-Erk, and p-CREB. GAPDH was used as a reference for normalization. n = 4. The data is presented as mean ± SEM. *P < 0.05, **P < 0.01, ***P < 0.001. P values are calculated by one-way ANOVA with Tukey's *post-hoc* test.

BDNF activates TrkB receptors and induces intracellular signaling pathways, such as PLCγ1/PKC, PI3K/AKT and MAPK/ERK pathway that trigger CREB activation, thereby promoting cell survival, axon growth of neurons, and synaptic plasticity 37. As compared with the normal control mice, the phosphorylation of PLCγ1, Akt, Erk and CREB were reduced in hippocampus or PFC of AD mice. MSC-EVs-anta significantly enhanced the phosphorylated levels of TrkB, Akt, Erk and CREB in hippocampus and PFC, but just enhanced the phosphorylated level of PLCγ1 in hippocampus. TrkB, Akt, Erk and CREB were slightly activated by MSC-EVs, MSC-EVs-nc or miR-206-3p antagomir (Figure 6A-C). These data suggested that MSC-EVs-anta effectively transport miR-206-3p antagomir to the AD brain and activated BDNF/TrkB pathway by upregulating BDNF.

### MSC-EVs-anta contribute to neurogenesis and restore dendritic length in the AD brain

**Figure 7:**
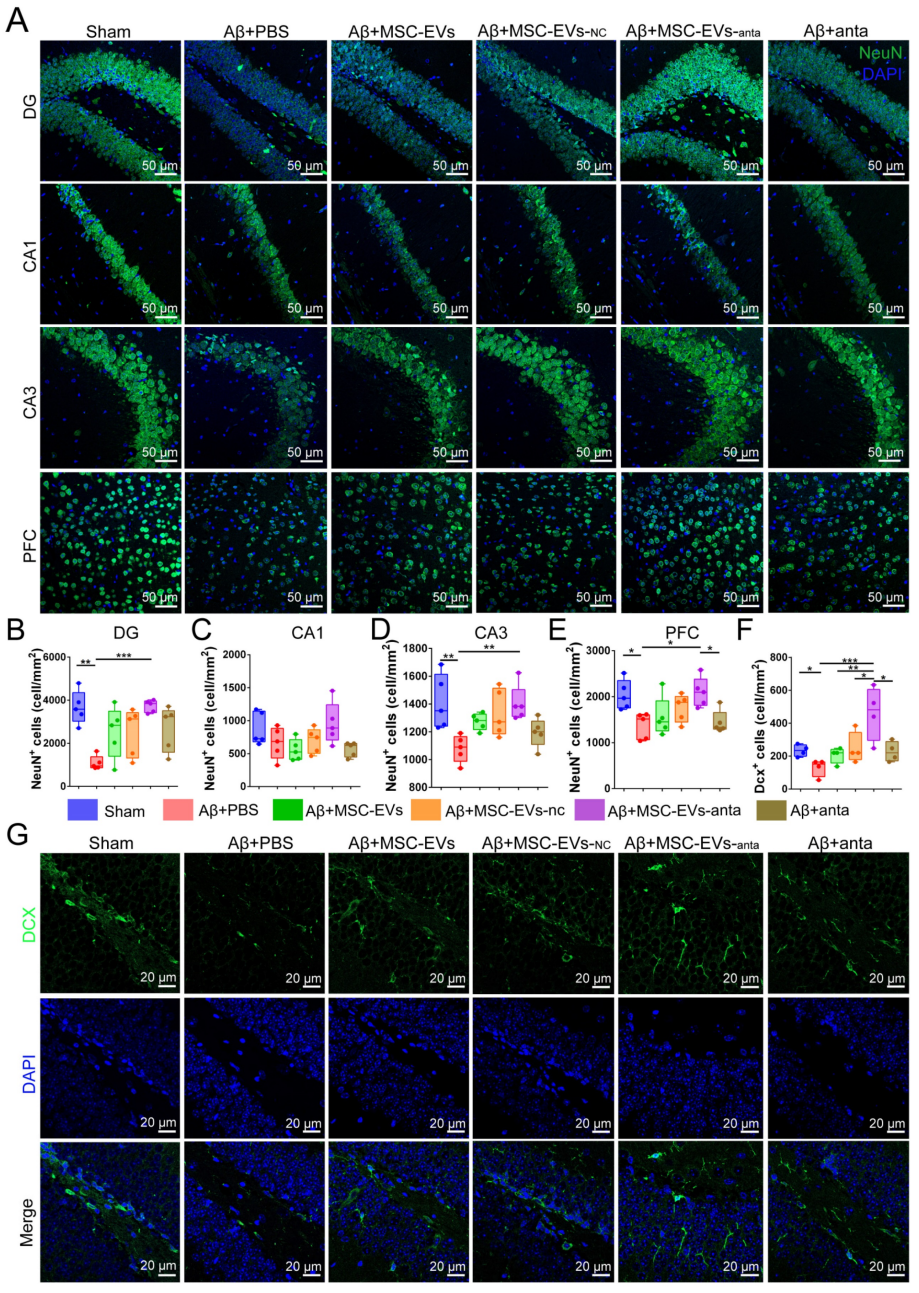
** MSC-EVs-anta contribute to neurogenesis in the AD brain. (A)** The representative images of NeuN^+^ cells in the hippocampus (DG, CA1 and CA3 regions) and PFC. Scale bar: 50 μm. **(B-E)** Quantification of the density of NeuN^+^ cells in the DG **(B)**, CA1 **(C)** and CA3 regions **(D)** and PFC **(E)**, n = 5.** (F-G)** Anti-Dcx immunostaining (green) indicated immature neurons in the DG region of the hippocampus, and quantitative analysis of density was performed, n = 5. The data is presented as mean ± SEM. **P < 0.01, ***P < 0.001. P values are calculated by one-way ANOVA with Tukey's *post-hoc* test.

The activation of BDNF/TrkB pathway contributes to neurogenesis 38. In our study, brain sections were immunolabeled for NeuN (mature neuron marker) and doublecortin (DCX, immature neuron marker). Immunofluorescence results showed that the number of NeuN-positive cells decreased in the hippocampal dentate gyrus (DG), CA3 area and PFC of AD mice, while not in CA1 area. However, intranasal delivery of MSC-EVs-anta reversed the reduction of NeuN-positive cells in AD mice. In AD mice treated with MSC-EVs, MSC-EVs-nc or miR-206-3p antagomir, the number of NeuN-positive cells also increased, but not significantly (Figure 7A-E). DCX-positive cells are distributed along the inner side of the granule layer in DG region. The number of DCX-positive cells decreased in AD mice, while intranasal delivery of MSC-EVs-anta sharply reversed the reduction, but MSC-EVs, MSC-EVs-nc or miR-206-3p antagomir had almost no impact on it (Figure 7F-G).

**Figure 8:**
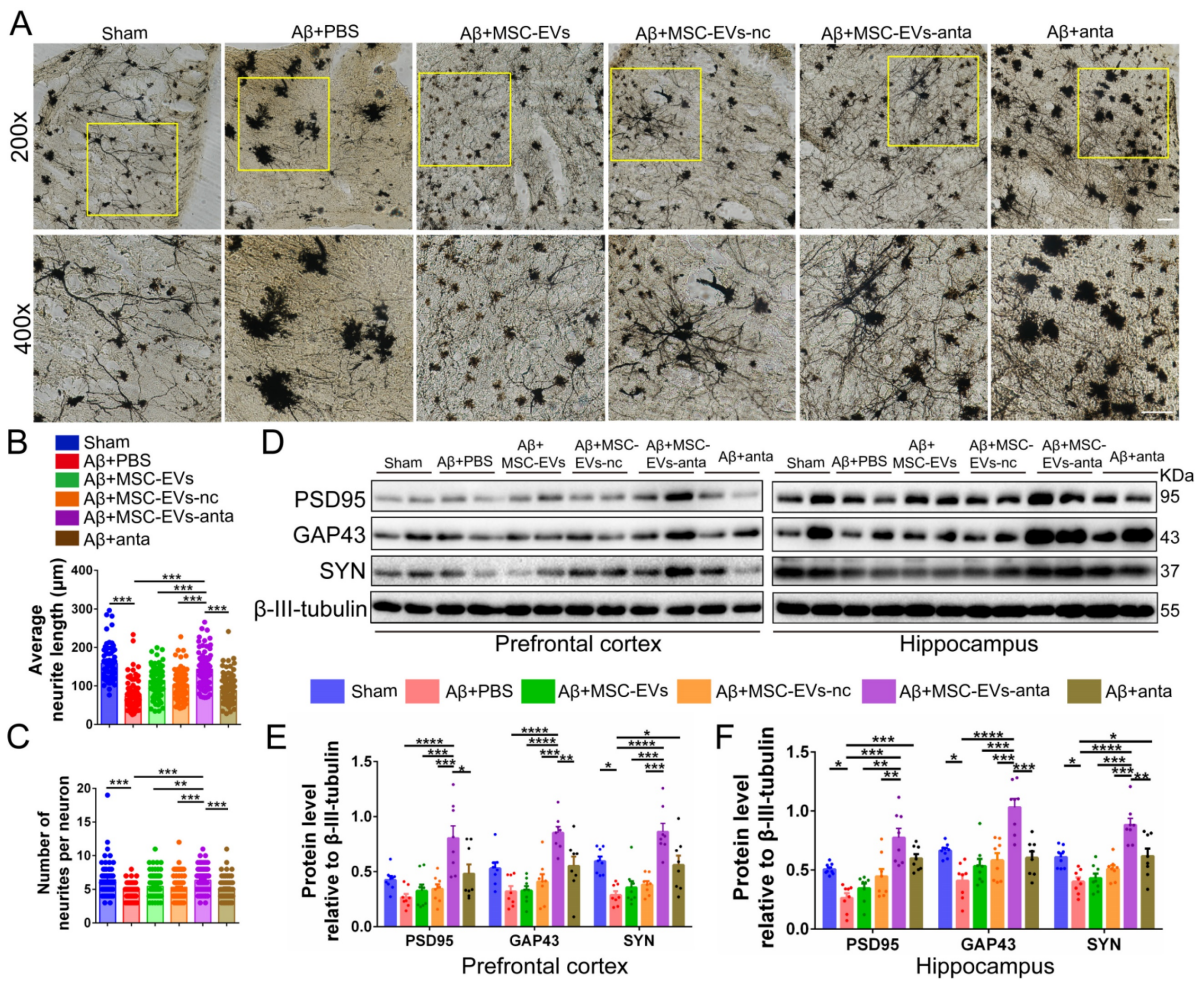
** MSC-EVs-anta restore dendritic length in the AD brain. (A)** The representative morphology of neurons with Golgi staining in the PFC of AD mice with intranasal administration of MSC-EVs-anta, MSC-EVs, MSC-EVs-nc or miR-206-3p antagomir. Scale bar: 100 μm. Average neurite length **(B)** and neurite number **(C)** analysis were conducted to evaluate the neuritic complexity, n = 62-102 neurons per group from 3 mice. **(D)** The representative Western blotting images of PSD95, GAP43 and SYN in the PFC and hippocampus of AD mice. **(E-F)** The quantification of PSD95, GAP43 and SYN in the PFC** (E)** and PSD95, GAP43 and SYN **(F)** in the hippocampus, n = 8. The data is presented as mean ± SEM. *P < 0.05, **P < 0.01, ***P < 0.001, ****P < 0.0001. P values are calculated by one-way ANOVA with Tukey's *post-hoc* test.

To further evaluate the effect of MSC-EVs-anta on the neural plasticity *in vivo*, we used Golgi staining to visualize the neural structure. The neurite number and length of cortical neurons were reduced in AD mice, but increased after treatment with MSC-EVs-anta. In AD mice treated with MSC-EVs, MSC-EVs-nc or miR-206-3p antagomir, the length of cortical neurons also increased, but still not significantly (Figure 8A-C). Additionally, we investigated the expression of PSD95, GAP43 and SYN in AD brain. MSC-EVs-anta significantly upregulated the levels of PSD95, GAP43 and SYN, which were downregulated in the PFC and hippocampus of AD mice. Although miR-206-3p antagomir treatment upregulated the expression of SYN, the upregulation was not as significant as MSC-EVs-anta (Figure 8D-F). These results demonstrate that MSC-EVs-anta has beneficial effects on neurogenesis and synaptic plasticity.

### MSC-EVs-anta alter the transcriptome and proteome expression profiles in the hippocampus of AD mice

**Figure 9:**
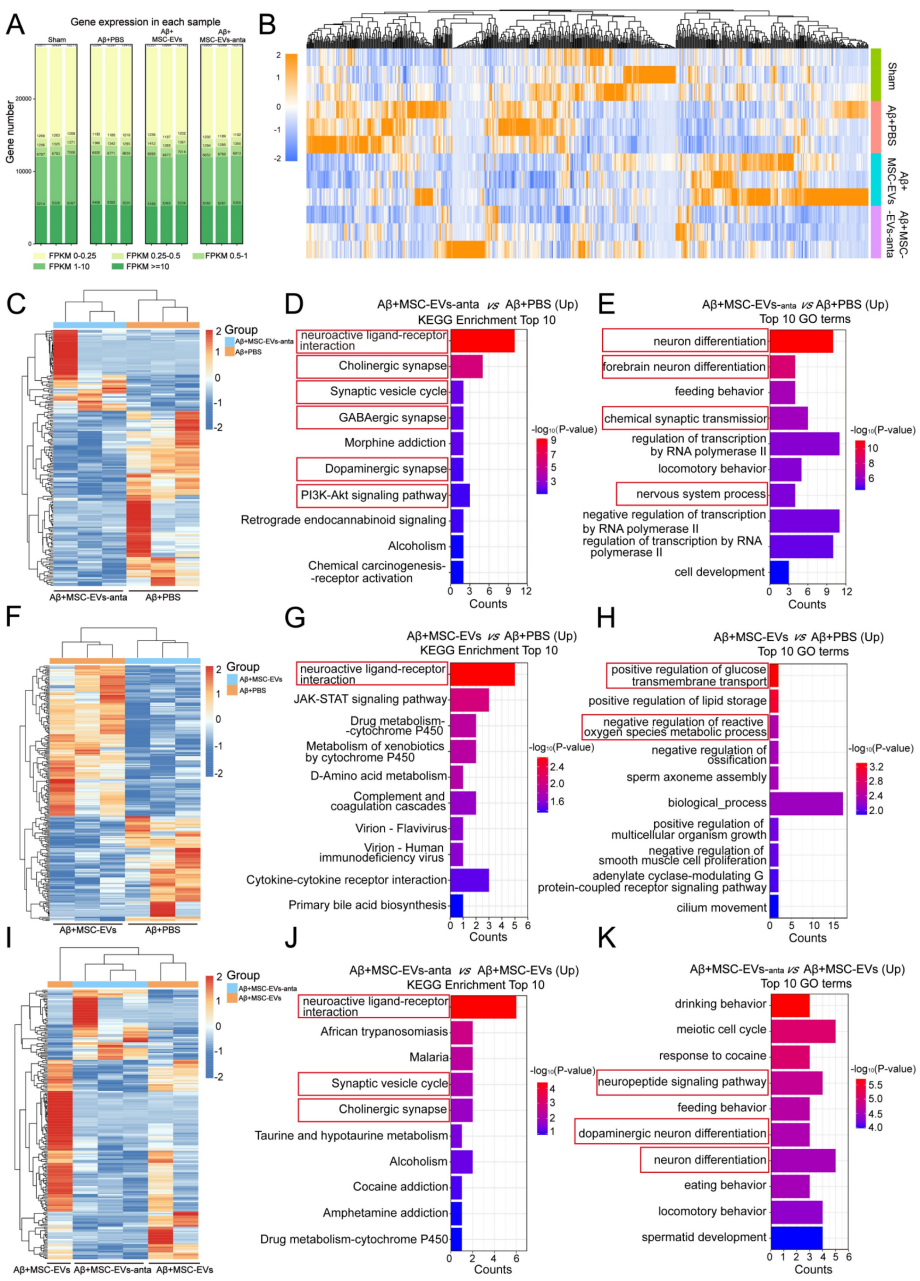
** Transcriptomic analysis in the hippocampus of AD mice with intranasal administration of MSC-EVs or MSC-EVs-anta. (A)** The distribution of expressed genes (FPKM values for each sample gene) in the hippocampus of AD mice using RNAseq after intranasal administration of PBS, MSC-EVs or MSC-EVs-anta. **(B)** Heatmap of the DEGs (log_2_ FC ≥ 2 or ≤ - 2; p-value < 0.05) among the four groups. **(C-K)** Heatmap of the DEGs, KEGG and GO analysis shows the top 10 terms enriched in upregulated DEGs. **(C-E)** MSC-EVs-anta administration compared with PBS treatment. **(F-D)** MSC-EVs administration compared with PBS treatment. **(I-K)** MSC-EVs-anta administration compared with MSC-EVs administration.

The transcriptome sequencing analysis on hippocampus was performed to disclose the mechanism underlying the improvement of MSC-EVs-anta in AD phenotype. The three replicates showed a high reproducibility with Pearson's correlation coefficient above 0.97 (Figure 9A and S5A). Compared with group Aβ+PBS, there were 234 DEGs in group Aβ+MSC-EVs; and there were 177 DEGs in group Aβ+MSC-EVs-anta. Compared with group Aβ+MSC-EVs, there were 199 DEGs in group Aβ+MSC-EVs-anta (Figure S5B). The DEGs in four groups were subsequently analyzed by hierarchical cluster and pathway enrichment analysis in each comparison (Figure 9B). Compared with group Aβ+PBS, Kyoto Encyclopedia of Genes and Genomes (KEGG) pathway and Gene Ontology (GO) terms analysis showed that both the upregulated DEGs in group Aβ+MSC-EVs-anta and those in group Aβ+MSC-EVs mainly enriched in signaling pathways related to neuron and synapse (Figure 9C-H, S6A, S6C, S6E, S7A-D and S7F-H). It is noteworthy that the number of enriched pathways in group Aβ+MSC-EVs-anta were more than that in group Aβ+MSC-EVs. Moreover, the upregulated DEGs in group Aβ+MSC-EVs-anta still enriched in signaling pathways related to neuron and synapse as compared with group Aβ+MSC-EVs (Figure 9I-K, S6G and S6I-M). Compared with group Aβ+PBS, KEGG pathway and GO terms analysis showed that the enriched signaling pathways in downregulated DEGs of group Aβ+MSC-EVs-anta or Aβ+MSC-EVs had no direct correlation with neuron or synapse (Figure S5C-H, S6B, S6D, S6F, S6H and S7E).

**Figure 10:**
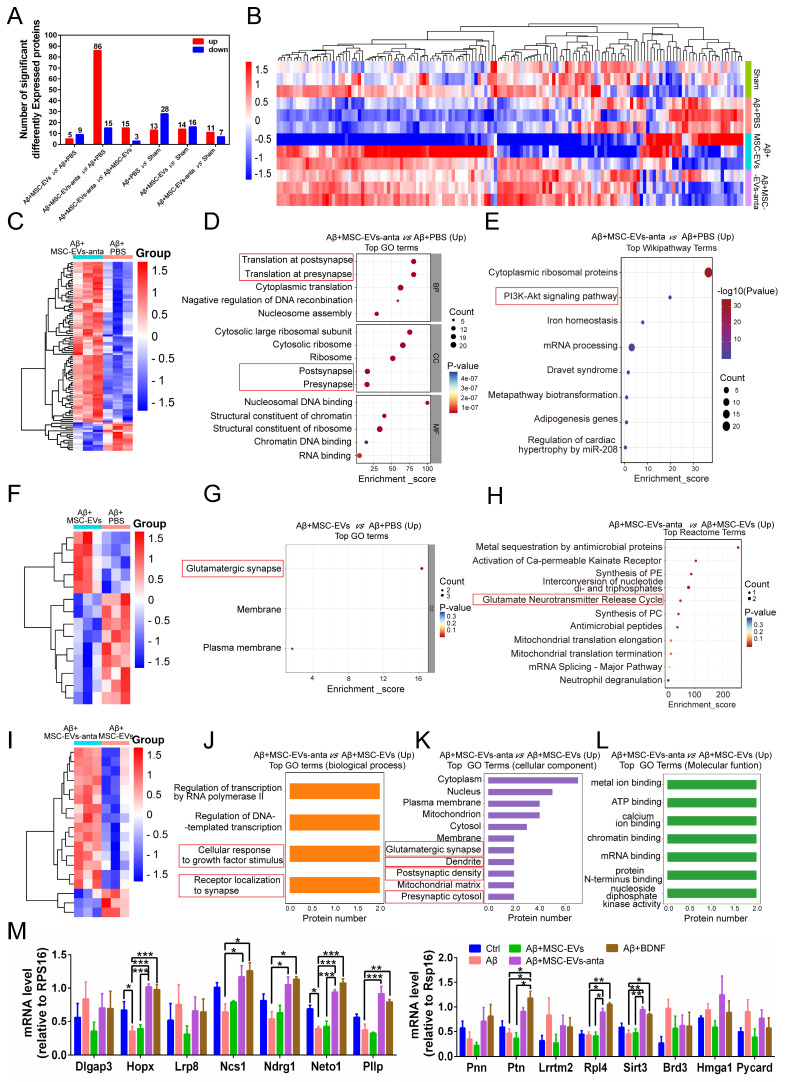
** Comparative proteomics and combined transcriptomics/proteomics in the hippocampus of AD mice. (A)** The histogram summarized the number of DEPs in the hippocampus using TMT (Tandem Mass Tag) labeling. **(B)** Proteomic clustering in Sham, PBS, MSC-EVs and MSC-EVs-anta groups. **(C)** Proteomic clustering comparison between group MCS-EVs-anta and group PBS. **(D-E)** Compared with group PBS, GO and Wikipathway analysis shows the top terms of enriched pathways in upregulated DEPs of group MCS-EVs-anta. **(F)** Proteomic clustering comparison between group MCS-EVs and group PBS. **(G-H)** Compared with group PBS, GO and Reactome analysis shows the top terms of enriched pathways in upregulated DEPs of group MCS-EVs. **(I)** Proteomic clustering comparison between group MCS-EVs-anta and group MCS-EVs. **(J-L)** Compared with group MCS-EVs, GO analysis shows the top terms of enriched pathways in upregulated DEPs of group MCS-EVs-anta. **(M)** Neurogenesis and inflammation-related genes, selected by combination of transcriptomics/proteomics analysis, were examined using qPCR. n = 3. The data is presented as mean ± SEM. *P < 0.05, **P < 0.01, ***P < 0.001. P values are calculated by one-way ANOVA with Tukey's *post-hoc* test.

For double confirming the DEGs, proteomics was employed to identify the differentially expressed proteins (DEPs) of hippocampal tissue in AD mice. A total of 3336 proteins were identified, and 136 DEPs were used for cluster analysis and the expression abundance was shown in the heatmap (Figure 10A-B). Undoubtedly, the mice treated with Aβ+MSC-EVs-anta showed more DEPs than the mice treated with Aβ+MSC-EVs compared with AD mice; on the contrary, the mice treated with Aβ+MSC-EVs-anta showed less DEPs than the mice treated with Aβ+MSC-EVs compared with sham mice (Figure 10A). After clustering and signal pathway enrichment analysis, the upregulated DEPs of group Aβ+MSC-EVs-anta were mainly enriched in postsynapse, presynapse and PI3K-Akt signaling pathway compared with group Aβ+PBS (Figure 10C-E and S8A-C). However, the upregulated DEPs of group Aβ+MSC-EVs only enriched in glutamatergic synapse pathway compared with group Aβ+PBS (Figure 10F-G). Moreover, many signaling pathways related to neuron and synapse were enriched in group Aβ+MSC-EVs-anta compared with group Aβ+MSC-EVs, including cellular response to growth factor stimulation, receptor localization to synapse, glutamatergic synapse, postsynaptic density, presynaptic cytosol and glutamate neurotransmitter release cycle (Figure 10H-L and S8D-F). Consistent with the transcriptomic analysis data, the enriched signaling pathways in downregulated DEPs of group Aβ+MSC-EVs-anta or Aβ+MSC-EVs had no direct correlation with neuron or synapse (Figure S9).

There were 15 differentially expressed proteins and genes (DEPGs) in group Aβ+MSC-EVs-anta compared with group Aβ+MSC-EVs or group Aβ+PBS (Figure S10). The upregulated DEPGs are involved in promoting neuronal growth, regulating synaptic remodeling, axonal growth and regulating hippocampal memory, such as NCS1, Neto1, Ndrg1, Pllp, Rpl4, Hopx and Ptn. Some of DEPGs are involved in regulating inflammatory responses, such as Sirt, Brd3, Hmgal and Pycard. Since miR-206-3p antagomir helps upregulate BDNF level, primary hippocampal cells were treated with exogenous BDNF as a positive control for MSC-EVs-anta. Compared with MSC-EVs, MSC-EVs-anta increased the mRNA levels of *Hopx*, *NCS1*, *Ndrg1*, *Neto1*, *Pllp*, *Ptn*, *Rpl4* and *Sirt3*, but not of *Dlgap3*, *Lrp8*,* Pnn*, *Lrrmt2*, *Brd3*,* Hmga1 and Pycard* in hippocampal cells damaged by Aβ (Figure 10M). Notably, MSC-EVs-anta had the same effects on the regulation of mRNA levels as exogenous BDNF, which suggests that MSC-EVs-anta targeted on BDNF to induce the following signaling pathways for neuroprotection.

## Discussion

Although MSCs have shown potential for promoting AD syndrome, MSC-based clinical translation is hindered by concerns, including tumorigenesis 23, vascular obstruction 39 and immune rejection 16. Interestingly, the use of MSC-secreted EVs can effectively avoid these issues. MSC-EVs display many advantages, such as high biocompatibility, stable storage, and the ability to cross the BBB, making them an excellent drug delivery vehicle 23, 40. More and more evidences suggest that loading therapeutic cargo into MSC-EVs is a promising strategy for AD therapy. EVs secreted by adipose-derived MSCs transfected with miR-22 show a favorable neuroprotection in AD mice by inhibiting pyroptosis 41. Treatment with SHP2-loaded MSC-EVs induce mitophagy of neurons to prevent apoptosis and rescue the loss of synapses in AD mice 42. Although these strategies have improved the efficacy of MSC-EVs, the complexity of endogenous factors still limits their application.

In the present study, we utilized physical electroporation to transfect miR-206-3p antagomir into MSC-EVs, thereby constructing MSC-EVs-anta. This method mitigates the risks associated with exogenous factors and maintains the stability of cargo. During electroporation, the applied electric field promotes the permeability of membrane, thereby inducing the formation of transient pores in EVs membrane. Previous study highlights the importance of voltage (V) as a critical parameter in electroporation 43. Thus, we evaluated the effects of different voltages on the loading efficiency of miR-206-3p antagomir in MSC-EVs. After electroporation at a pulse of 750 V, MSC-EVs showed the highest efficiency in loading miR-206-3p antagomir, far higher than the encapsulation efficiency of simple incubation. This indicated that electroporation with 750 V did not cause RNA leakage from native EVs. It is reported that a high voltage (1000 V) disrupts the pore structure of EVs 35. We also found that the amount of antagomir loaded into EVs decreased as the voltage increased to 1000 V. Alterations in the membrane composition and leakage of EVs content may affect EVs biological activity and target cell uptake. After electroporation, the expression of EV membrane proteins CD63, CD81 and CD9, as well as the integrity of membrane remain unchanged. The tetraspanin proteins CD81 and CD9 play a role in cell binding and uptake of EVs 44-46. Our results also showed that there was no significant change in the levels of CD63, CD81 and CD9 in MSC-EVs subjected to electroporation. Besides, the size and quantity of MSC-EVs were unaffected. These findings confirm the feasibility of electroporation for constructing miR-206-3p antagomir-loaded MSC-EVs.

AD patients showed high miR-206-3p levels, which resulted in AD progression by inhibition of BDNF expression. We have demonstrated that brain injection of miR-206-3p antagomir into the hippocampus has therapeutic effects on AD mice 31. However, intracerebral injection is highly invasive and not suitable for long-term treatment. Although miR-206 antagomir can be delivered into brain by nasal administration 47, the limitation still involves low absorption rates, susceptibility to degradation, and potential risks of drug overdose. Notably, we effectively overcome these challenges by loading miR-206-3p antagomir into MSC-EVs. After intravenously injection, MSC-EVs mainly accumulate in the liver and spleen, rather than the brain 48. As a non-invasive route of administration, intranasal delivery has multiple advantages of painlessness, rapid onset of action, effective brain targeting, and reduced systemic side-effects 49-51. Recently, the results of phase I/II clinical trial demonstrated that there were no adverse reactions in 24 healthy subjects receiving intranasal administration of adipose-derived MSC-EVs 52.

We employed intranasal administration to deliver engineered MSC-EVs (MSC-EVs-anta) and found that MSC-EVs could enter the brain and were mainly taken up by neurons. Intranasal administration of MSC-EVs (2×10^9^ EVs per nostril) can reduce Aβ deposition in the brains of 5×FAD mice, while intravenous injection of EVs (100 µg for each mouse) does not have the same effect 19, 42. In the present study, MSC-EVs and MSC-EVs-nc did not reduce Aβ accumulation, possibly due to the low dosage of EVs (5×10^8^ EVs per nostril) administered intranasally. MSC-EVs significantly increased the average length of cortical neuron axons at a dosage of 3×10^8^ particles/mL 53. Compared with this, the low concentration of MSC-EVs and MSC-EVs-nc (~1×10^8^ particles/mL) used in our experiment did not promote neuronal dendrite growth *in vitro*. Interestingly, treatment with MSC-EVs-anta contributed to the reduction of Aβ deposition, acceleration of neurogenesis and maintaining synaptic plasticity, thereby restoring the learning and memory abilities in AD mice. The therapeutic effect of MSC-EVs-anta is superior to MSC-EVs or miR-206-3p antagomir, highlighting the advantages of engineered MSC-EVs.

Next, we investigated the mechanism underlying the attenuation of AD symptoms by MSC-EVs-anta. MSC-EVs alone were unable to suppress the expression of miR-206-3p in the AD brains, while MSC-EVs-anta significantly downregulated the levels of miR-206-3p, which attributed to the encapsulated miR-206-3p antagomir. Compared to direct administration of miR-206-3p antagomir, treatment with MSC-EVs-anta demonstrated superior efficacy in reducing miR-206-3p levels. This suggests that MSC-EVs have excellent cargo carrying capacity and can protect miR-206-3p antagomir from degradation. BDNF, as a target of miR-206-3p, plays an important role in promoting neurogenesis and reducing Aβ deposition 31, 54. However, the challenges of exogenous BDNF administration involves poor BBB permeability and rapid degradation. Interestingly, MSC-EVs-anta significantly increased BDNF level and activated the downstream signaling pathways such as Akt, Erk and CREB. Activation of the BDNF/TrkB pathway promotes neuronal survival, neurogenesis and synaptic plasticity, which supports the conclusion that MSC-EVs-anta increase NeuN and DCX-positive cells, and upregulate the expression of PSD95, SYN and GAP43. The data of two-omics analysis further confirmed that the proteins and genes significantly modulated by MSC-EVs-anta were primarily enriched in neurons and synapses, which is consistent with the consequence induced by BDNF.

Chemical transfection is a commonly used method for loading therapeutic biomolecules into EVs, such as proteins and nucleic acids. Due to its relatively low transfection efficiency and safety, it still remains controversial in clinical applications 55. In the present study, the preparation of MSC-EVs-anta does not involve the use of viral vectors, transfection regents, or potentially hazardous chemicals, which further enhances its clinical applicability. A recent study has demonstrated that acoustic shock waves can achieve an efficiency of over 70% when loading siRNA into EVs 36. Although our loading efficiency has not matched the above level, our method has great potential in clinical applications, characterized by low requirements for equipment, low costs, simple process and good repeatability. Microfluidics has been employed to enhance drug delivery due to its precise control over fluids and electric fields 56, which may be a promising technology for improving loading efficiency of EVs in the future. However, there are currently no standard guidelines for isolation and quality control of EVs in clinical applications. Therefore, further investigation and exploration are necessary to ensure the safe translation of MSC-EVs therapy from the laboratory to the bedside.

In conclusion, MSC-EVs loaded with miR-206-3p antagomir were successfully constructed using electroporation technology. MSC-EVs-anta showed promising therapeutic effects in AD mice, including improving cognitive deficits, promoting hippocampal neurogenesis and synaptic plasticity, and reducing Aβ burden. MSC-EVs-anta significantly upregulated BDNF in AD mice, and activated the BDNF/TrkB signaling pathway. The differentially expressed proteins and genes significantly regulated by MSC-EVs-anta were primarily enriched in the pathways involved in neurogenesis and synapse. Given the advantages of MSC-EVs and the targeting strategy of BDNF in the treatment of AD, these findings highlight the potential for future clinical applications.

## Supplementary Material

Supplementary figures.

## Figures and Tables

**Table 1:** The primers used for qPCR

Rat-F-Neto1	TTCCTGGAGGGTCATGCAAC	Rat-F-Ptn	GCCCTGCACTTTGCAACAA
Rat-R-Neto1	CGCGCCACCTCTGATATCTT	Rat-R-Ptn	CGACGTTGCTGCTGGTATTG
Rat-F-Ncs1	GGTGTCGGGCACACAGAATA	Rat-F-Lrp8	TTCAGAGAAGATCACGGCCC
Rat-R-Ncs1	ACCTACCCAGCCCCTTAGTT	Rat-R-Lrp8	GGTGCCCAGTTATGGTTGCT
Rat-F-Dlgap3	GGATGGCGAGTGGTTCATCA	Rat-F-Sirt3	GCCCAATGTCGCTCACTACT
Rat-R-Dlgap3	AGAAACCGGCCAGGTCCAA	Rat-R-Sirt3	GCAAAAGGCTCCACCCGTAT
Rat-F-Ndrg1	TTCCTTGCTGGTTAGGTCTGTG	Rat-F-Brd3	AGCCAGAATAAGGAAGGAAGGG
Rat-R-Ndrg1	GGGTTCCACACAGAGTGACAT	Rat-R-Brd3	CATCCTCTGGCAGCTTAGTCA
Rat-F-Pnn	GGAAAGCAAAGGAACCGACG	Rat-F-Hmga1	TTACCGAGTACCCCACGCTA
Rat-R-Pnn	AGAAGCCGCAGTTCTGTCTG	Rat-R-Hmga1	AGGCTGGGACAAATACTGGC
Rat-F-Lrrtm2	TGGTGATCGGCGGTAATCAG	Rat-F-Pycard	TTGGCCTAAGCAGATGACTTC
Rat-R-Lrrtm2	TTCAGTCGAGGTTGTCAGGC	Rat-R-Pycard	GCTGTCAAGTTTTCAAGAGCG
Rat-F-Pllp	CCTTGATTGCTGACACCCCA	Rat-F-Rps16	AAGTCTTCGGACGCAAGAAA
Rat-R-Pllp	AGACCAGTAACACCAACGGC	Rat-R-Rps16	TTGCCCAGAAGCAGAACAG
Rat-F-Rpl4	CTACAACCTCCCCATGCACA	rno-miR-206-3p	Art.No. RmiRQP0308
Rat-R-Rpl4	ATCTTCTTGCGTGGTGCTCG	mmu-miR-206-3p	Art.No. MmiRQP0308
Rat-F-Hopx	CTCTCCATCCTTAGCCAGACG	RSnRNAU6	Art.No. MmiRQP9003
Rat-R-Hopx	GTCCGTGACCGATCTGCATT	MSnRNAU6	Art.No. MmiRQP9002

## Abbreviations

AD: Alzheimer's disease
MSC: Mesenchymal stem cells
EVs: Extracellular vesicles
MSC-EVs: MSC-derived EVs
MSC-EVs-nc: MSC-EVs containing antagomir NC
MSC-EVs-anta: MSC-EVs containing miR-206-3p antagomir
BBB: Blood-brain barrier
BDNF: Brain-derived neurotrophic factor
GAP43: Growth-associated protein 43
PSD95: Post-synaptic density-95
SYN: Synaptophysin
