# Supplementary material for: Intranasal delivery of engineered extracellular vesicles loaded with miR-206-3p antagomir ameliorates Alzheimer's disease phenotypes
Source: Theranostics. 2024 Nov 4;14(19):7623–44. doi: 10.7150/thno.103596 (PMC11626949; doi:10.7150/thno.103596)
Supplement: Supplementary file 1 — Supplementary figures. [file thnov14p7623s1.pdf]

Supplementary Materials for

Intranasal delivery of engineered extracellular vesicles loaded with miR-206-3p antagomir ameliorates Alzheimer's disease phenotypes

Dong Peng[a,b,d], Tingting Liu[a,b], Huahui Lu[a,b], Lei Zhang[a,b], Hongxia Chen[a,b,c], Yadong Huang[a,b,c], Bo Hu[d*], Qihao Zhang[a,b,c*]

[a] State Key Laboratory of Bioactive Molecules and Druggability Assessment, Guangdong Basic Research Center of Excellence for Natural Bioactive Molecules and Discovery of Innovative Drugs, College of Life Science and Technology, Jinan University, Guangzhou 510632, China;

[b] Department of Cell Biology & Institute of Biomedicine, College of Life Science and Technology, Jinan University, Guangzhou 510632, China;

[c] Guangdong Provincial Key Laboratory of Bioengineering Medicine, National Engineering Research Center of Genetic Medicine, Jinan University, Guangzhou 510632, China;

[d] Department of Laboratory Medicine, Third Affiliated Hospital of Sun Yat-sen University, Guangzhou 510630, China.

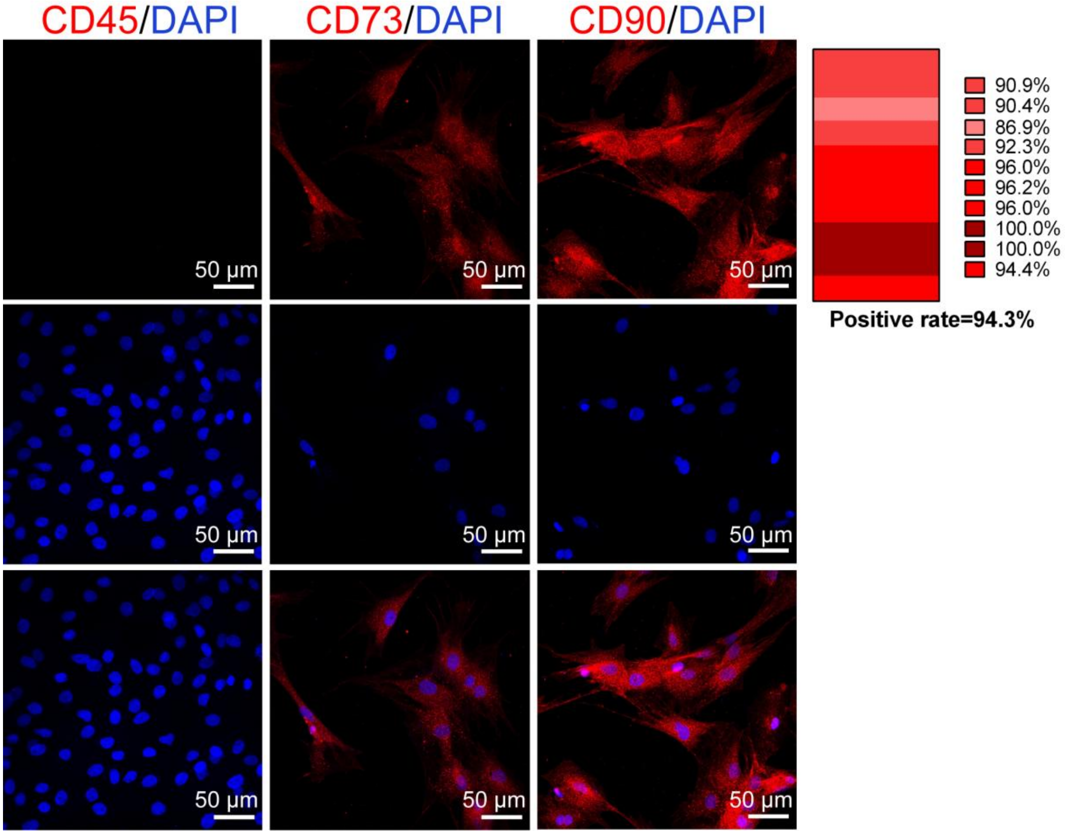

Figure S1 Identification of MSCs using surface markers. Immunostaining of MSCs disclosing negative staining of hematopoietic stem cell marker CD45 and positive staining for CD73 and CD90, which are the characteristics of MSCs. (Scale bar: 50 μm).

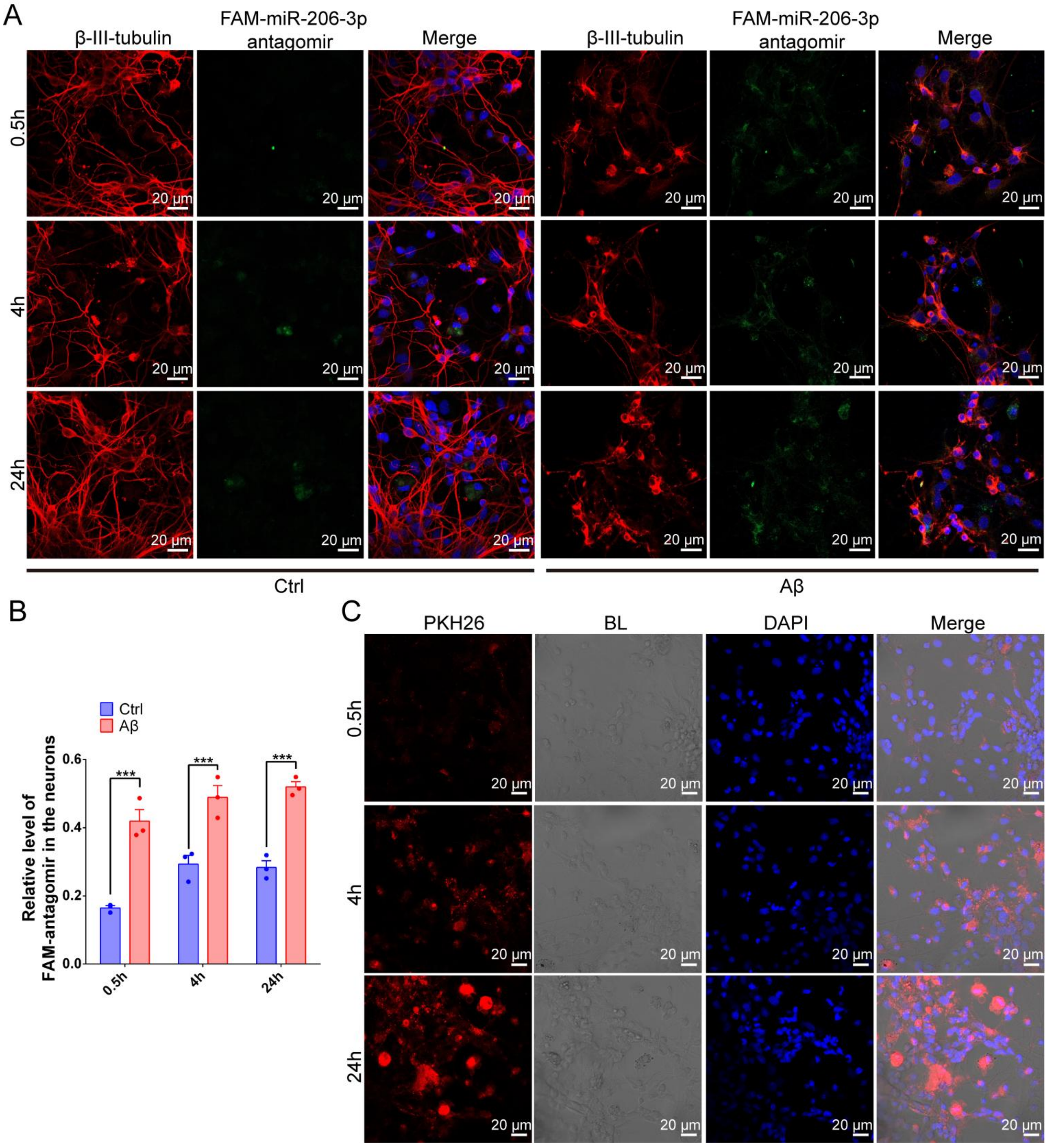

Figure S2 The uptake of FAM-miR-206-3p antagomir or PKH26-labeled MSC-EVs by primary cortical neurons were evaluated. (A) Under the conditions with or without Aβ damage, the primary cortical neurons were incubated with FAM-miR-206-3p antagomir for 0.5, 4 and 24 hours. The distribution of the FAM-miR-206-3p antagomir was measured using confocal microscopy. (Scale bar: 20 μm). **(B)** Quantified levels of FAM-miR-206-3p antagomir in neurons, n = 3. **(C)** Primary cortical neurons were incubated with PKH26-labeled MSC-EVs for 0.5 h, 4 h, and 24 h. The internalization of the

MSC-EVs was visualized using confocal microscopy. (Scale bar: 20 μm). The data are presented as mean ± SEM. ***$P < 0.001$. P values are calculated by Student's t-test.

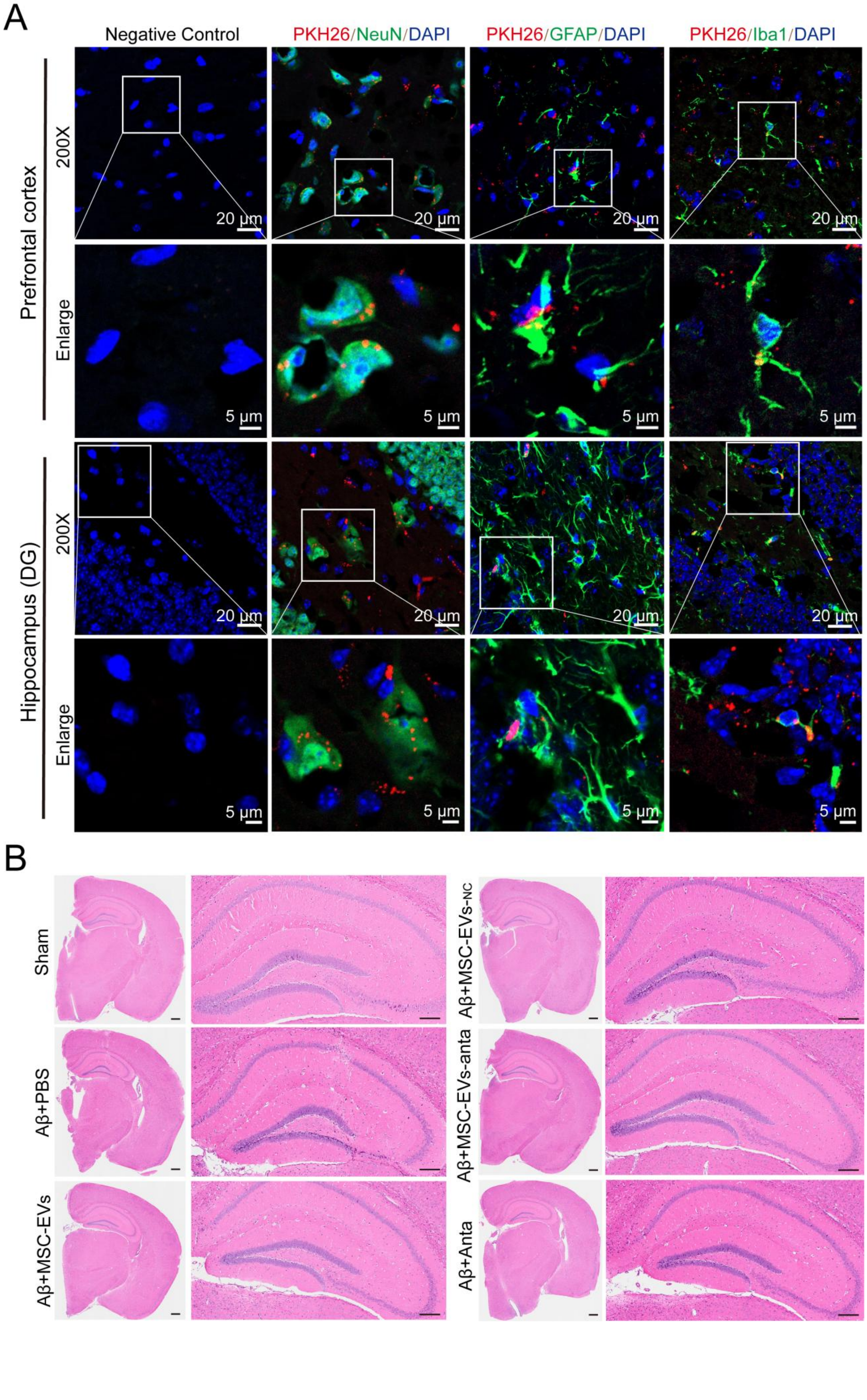

Figure S3 MSC-EVs distribution and histopathological analysis in the brain after intranasal administration. **(A)** In brain slices, double immunostaining revealed that most NeuN-positive neurons, as well as some Iba1-positive microglia and GFAP-positive astrocytes, contained PKH26-labeled MSC-EVs in the hippocampal DG and PFC. The negative control mice were administered an equivalent volume of PBS. (Scale bars: 20 μm). **(B)** The brain histomorphology of AD mice After intranasal administration of PBS, MSC-EVs, MSC-EVs-nc, MSC-EVs-anta and miR-206-3p antagomir, (H.E. staining. scale bar: 50 μm).

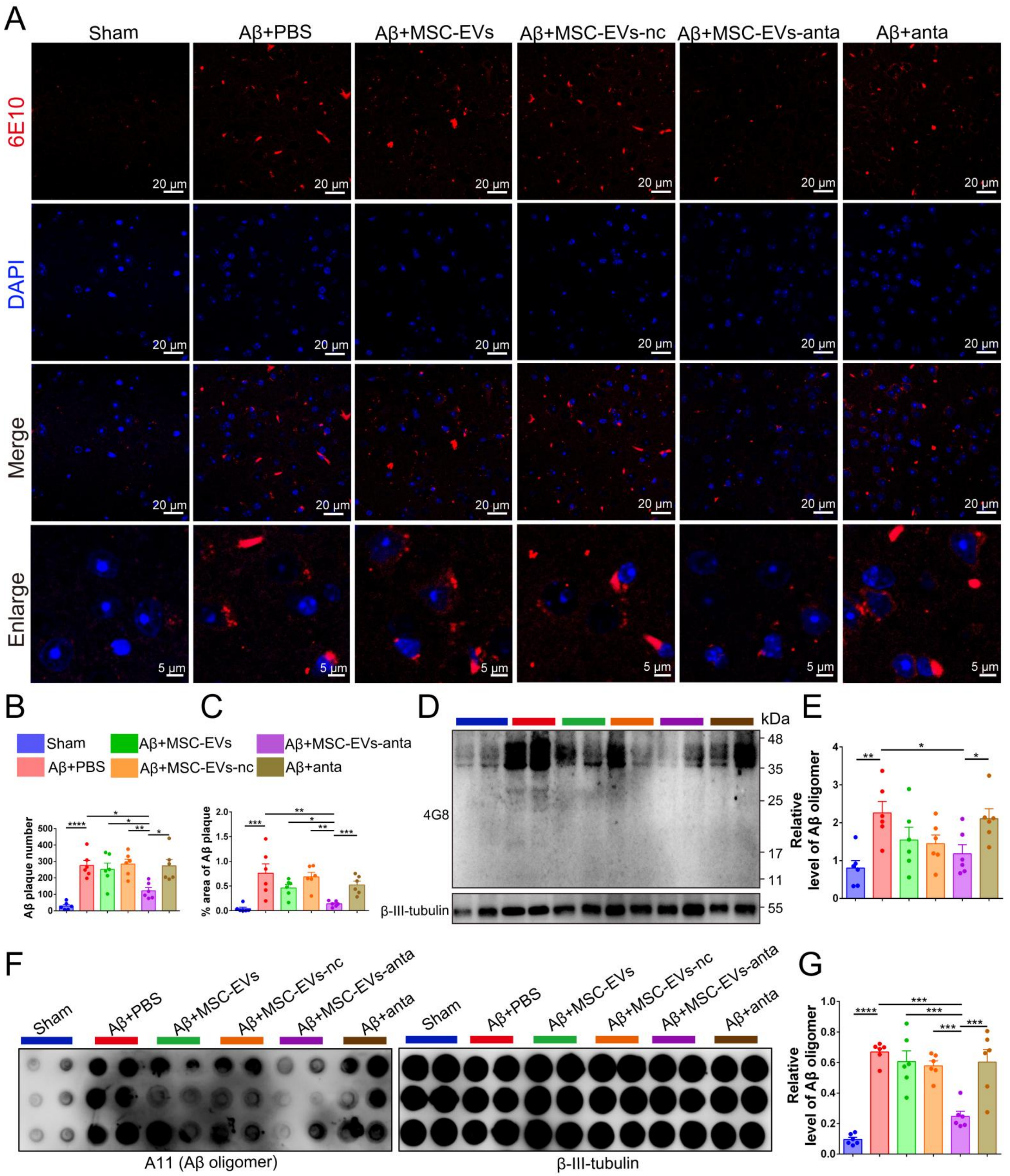

Figure S4 MSC-EVs-anta reduce Aβ burden in the PFC of AD mouse brain. (A) Aβ plaque in the PFC was stained by immunofluorescence. **(B-C)** The number and area of Aβ plaque are quantified by image-pro plus software, n = 6. **(D)** The representative immunoblots of Aβ in the PFC, and **(E)** quantified expression of Aβ normalized to β-III-tubulin, n = 6. **(F)** Aβ oligomers in the PFC were detected by dot

blotting. **(G)** Relative levels of Aβ oligomers, n = 6. The data is presented as mean ± SEM. *$P < 0.05$, **$P < 0.01$, ***$P < 0.001$, ****$P < 0.0001$. P values are calculated by one-way ANOVA with Tukey's *post-hoc* test.

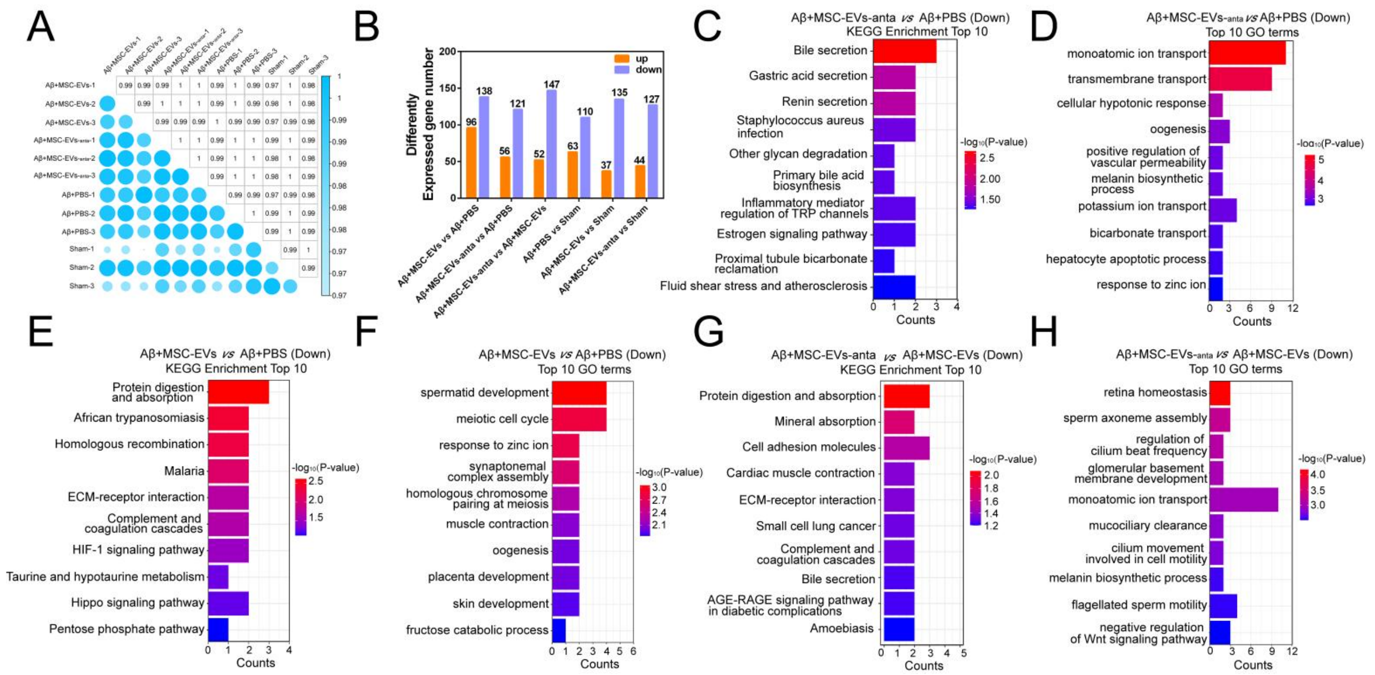

Figure S5 GO enrichment analysis of the differentially expressed genes. (A) Correlation coefficients among sequencing samples. **(B)** The number of DEGs in the hippocampus of AD mice using RNAseq. **(C-F)** KEGG and GO analysis shows top 10 terms enriched in downregulated DEGs. **(C-D)** MSC-EVs-anta administration compared with PBS treatment. **(E-F)** MSC-EVs administration compared with PBS treatment. **(G-H)** KEGG and GO enrichment analysis of downregulated DEGs in group MSC-EVs-anta compared to that in the group MSC-EVs.

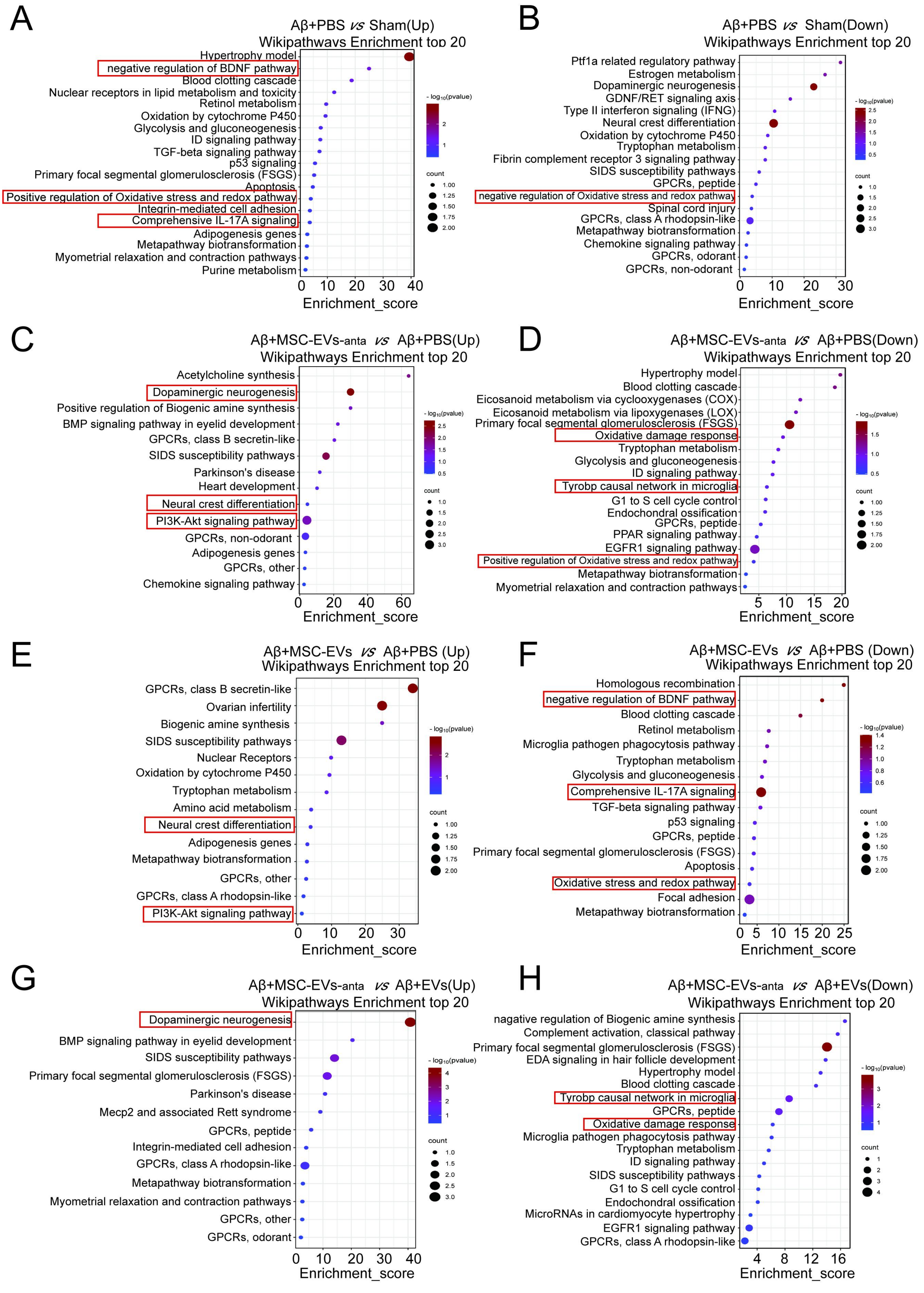

Figure S6 Wikipathway enrichment analysis of the differentially expressed genes. (A-F) Compared to the group PBS, Wikipathway analysis shows the top 20 terms enriched in upregulated DEGs and downregulated DEGs of Sham **(A-B)**, MSC-EVs-anta **(C-D)** and MSC-EVs groups **(E-F)**. **(G-H)** Wikipathway analysis of DEGs in group MSC-EVs-anta compared to that in the group MSC-EVs.

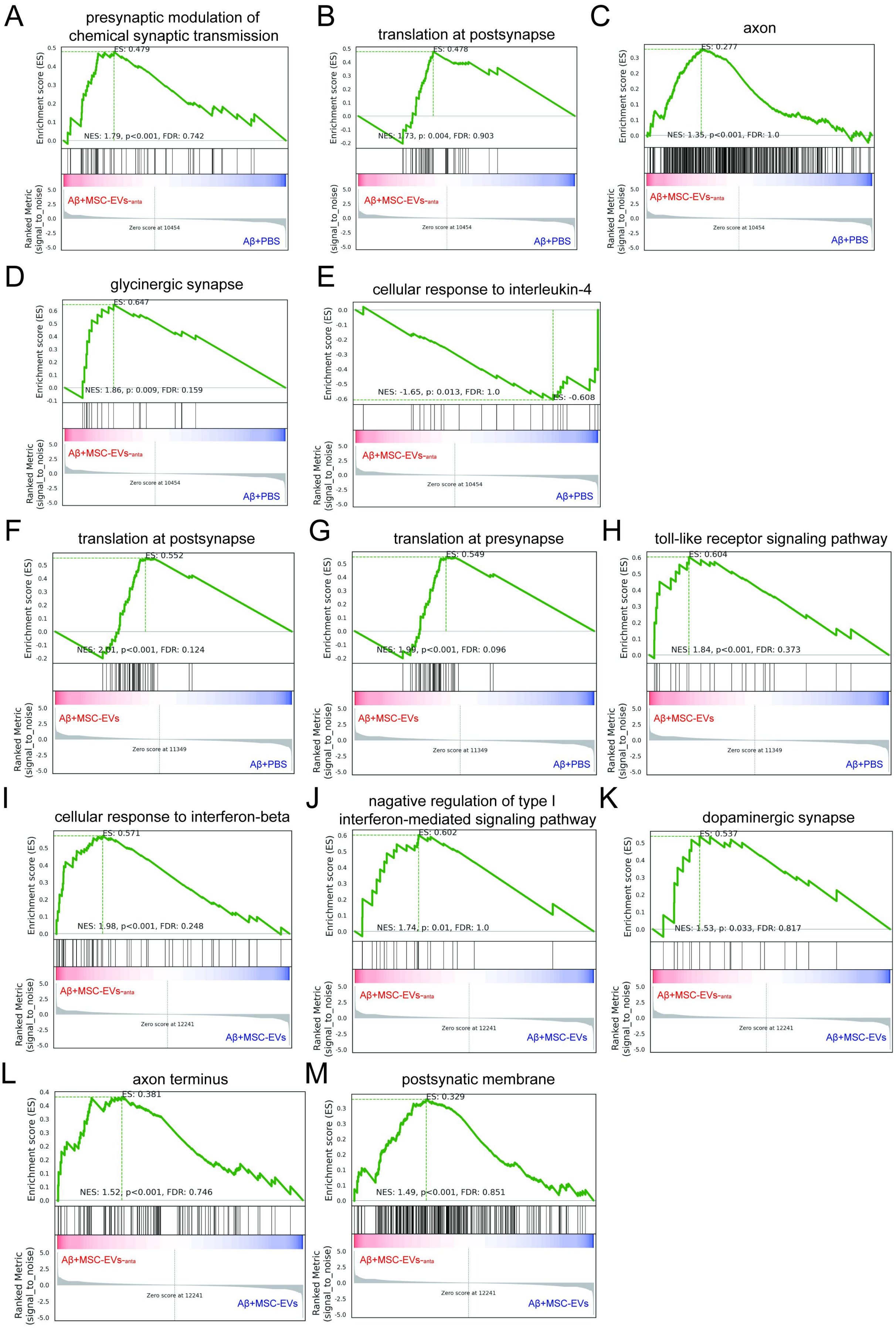

Figure S7 Gene Set Enrichment Analysis (GSEA) was conducted on the DEGs. (A-H) GSEA

revealed the enriched pathways for upregulated and downregulated DEGs in the group MSC-EVs-anta **(A-E)** or group MSC-EVs **(F-H)** compared to the group PBS, respectively. **(I-M)** GSEA revealed the enriched pathways for upregulated DEGs in the group MSC-EVs-anta compared to the group MSC-EVs.

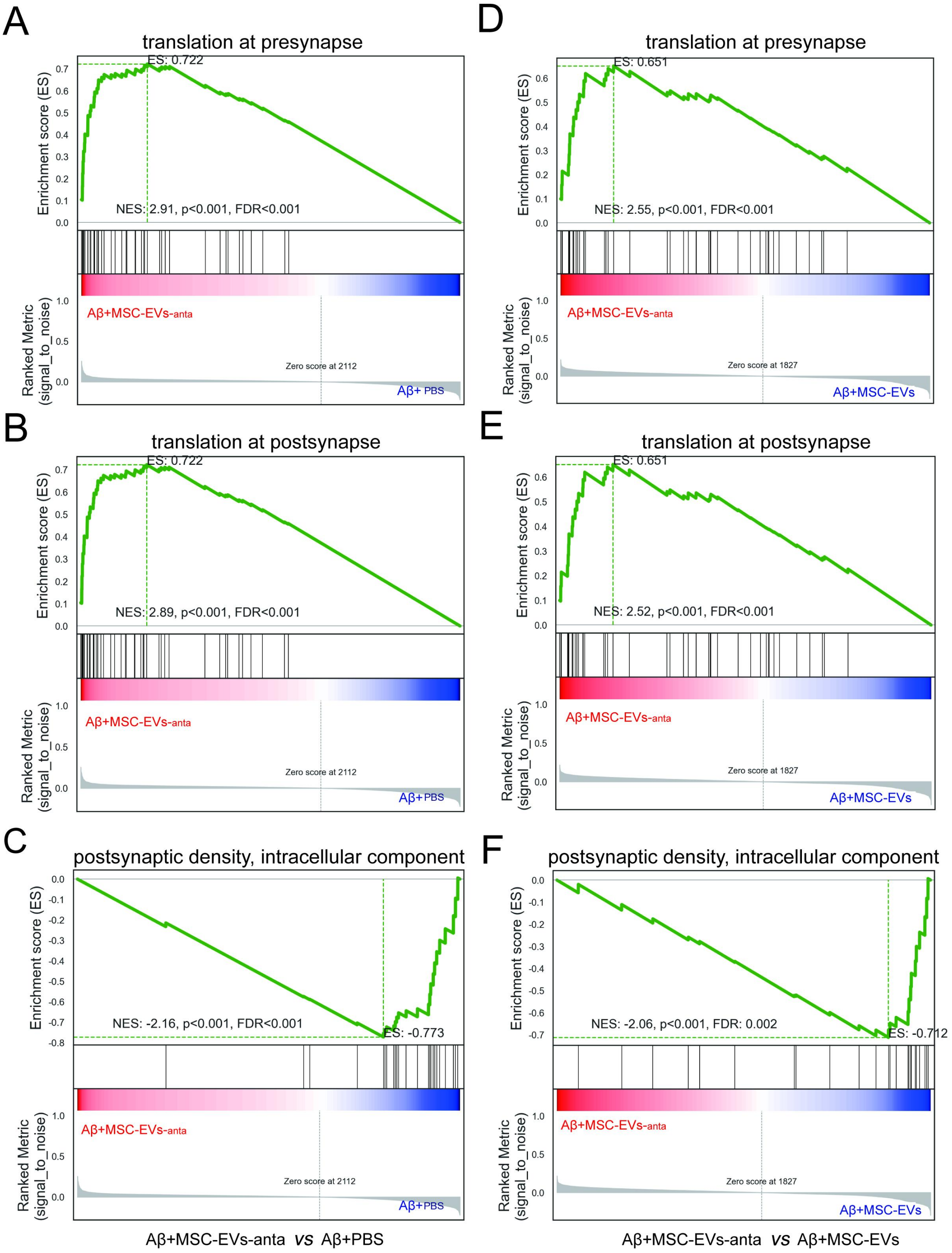

Figure S8 Gene Set Enrichment Analysis (GSEA) was conducted on the DEPs. (A-C) GSEA revealed the enriched pathways for upregulated and downregulated DEPs in the group MSC-EVs-anta compared to the group PBS. **(D-F)** The GSEA of upregulated and downregulated DEPs in the group MSC-EVs-anta

compared to group MSC-EVs.

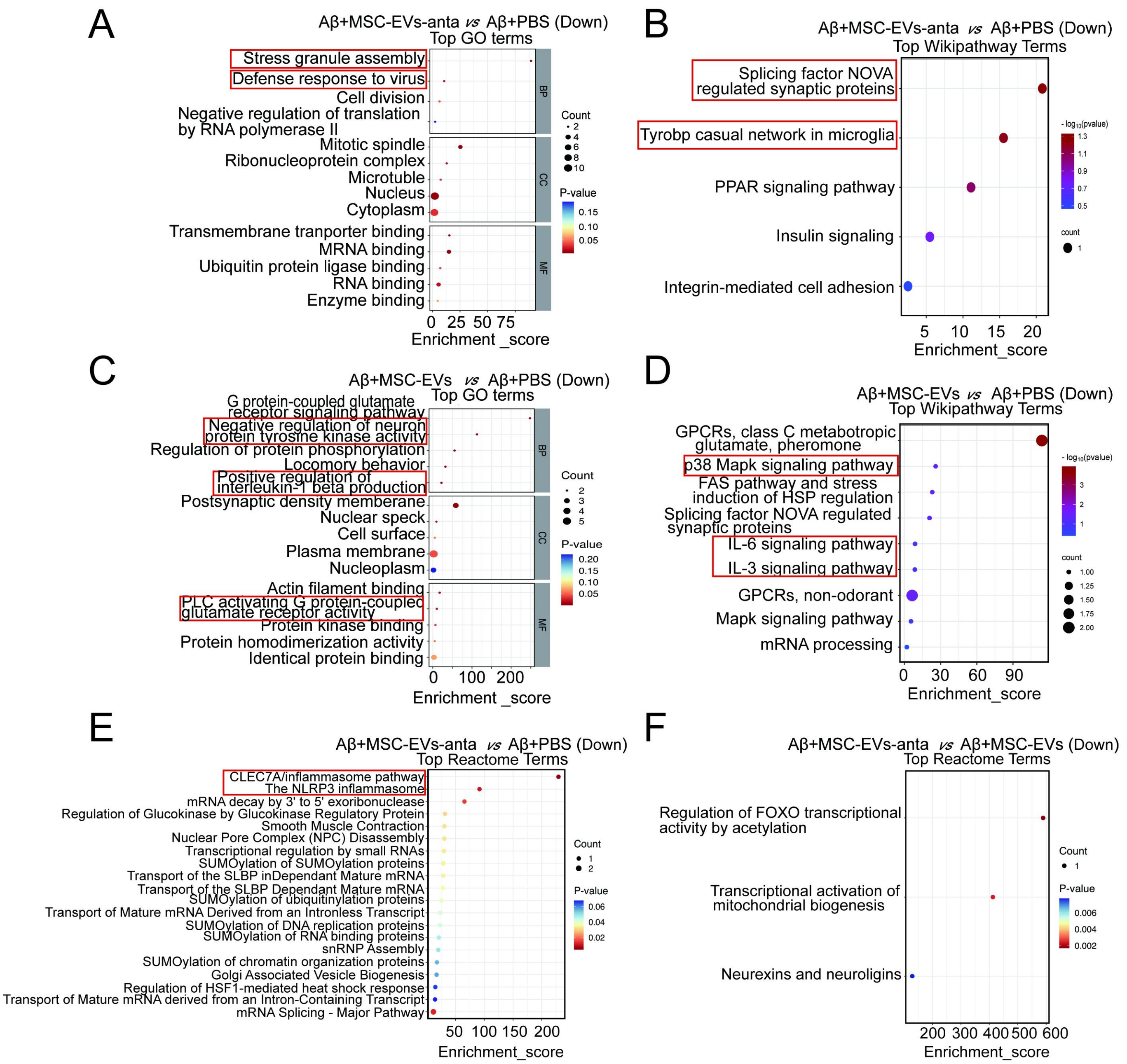

Figure S9 GO, Wikipathway enrichment and reactome terms analysis of the downregulated DEPs. (A-D) GO and **Wikipathway** analysis shows top terms from downregulated DEPs in group MSC-EVs-anta **(A-B)** and MSC-EVs **(C-D)** compared to the group PBS. **(E-F)** Bubble plot displays the enriched Reactome terms for downregulated proteins in group MSC-EVs-anta compared with group PBS **(E)**, and in group MSC-EVs-anta compared with group MSC-EVs **(F)**, respectively.

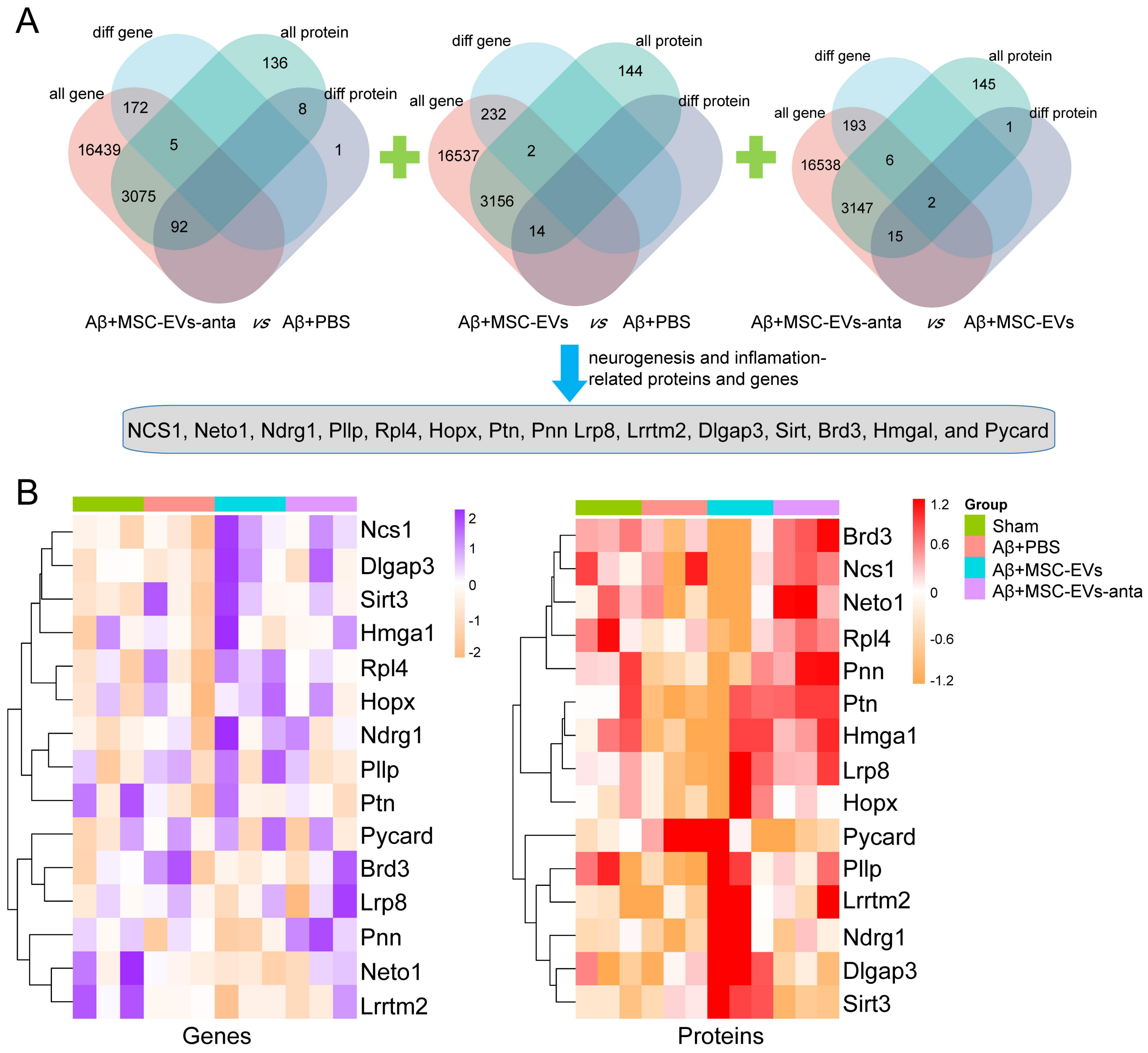

Figure S10 Integrated analysis of proteomics and transcriptomics. (A) Venn diagram analysis of the intersection between differentially expressed proteins and corresponding genes with consistent changes in group MSC-EVs-anta and MSC-EVs compared to the group PBS, and in group MSC-EVs-anta compared to group MSC-EVs. Through screening based on their relevance to neural growth and inflammation-related functions, a total of 15 molecules were obtained. **(B)** Transcriptomics and proteomic clustering of 15 molecules in PBS, Sham, MSC-EVs, and MSC-EVs-anta groups.
